# Seasonal Alterations in Organic Phosphorus Metabolism Drive the Phosphorus Economy of Annual Growth in *F. sylvatica* Trees on P-Impoverished Soil

**DOI:** 10.3389/fpls.2018.00723

**Published:** 2018-06-06

**Authors:** Florian Netzer, Cornelia Herschbach, Akira Oikawa, Yozo Okazaki, David Dubbert, Kazuki Saito, Heinz Rennenberg

**Affiliations:** ^1^Chair of Tree Physiology, Institute of Forest Sciences, Albert-Ludwigs-University Freiburg, Freiburg, Germany; ^2^Ecosystem Physiology, Institute of Forest Sciences, Albert-Ludwigs-University Freiburg, Freiburg, Germany; ^3^Metabolomics Research Group, RIKEN Center for Sustainable Resource Science, Yokohama, Japan; ^4^Graduate School of Pharmaceutical Sciences, Chiba University, Chiba, Japan; ^5^College of Science, King Saud University, Riyadh, Saudi Arabia

**Keywords:** phospholipids, metabolome, phosphorus nutrition, annual growth, whole plant nutrition

## Abstract

Phosphorus (P) is one of the most important macronutrients limiting plant growth and development, particularly in forest ecosystems such as temperate beech (*Fagus sylvatica*) forests in Central Europe. Efficient tree internal P cycling during annual growth is an important strategy of beech trees to adapt to low soil-P. Organic P (P_org_) is thought to play a decisive role in P cycling, but the significance of individual compounds and processes has not been elucidated. To identify processes and metabolites involved in P cycling of beech trees, polar-metabolome and lipidome profiling was performed during annual growth with twig tissues from a sufficient (Conventwald, Con) and a low-soil-P (Tuttlingen, Tut) forest. Autumnal phospholipid degradation in leaves and P export from senescent leaves, accumulation of phospholipids and glucosamine-6-phosphate (GlcN6P) in the bark, storage of N-acetyl-D-glucosamine-6-phosphate (GlcNAc6P) in the wood, and establishing of a phospholipid “start-up capital” in buds constitute main processes involved in P cycling that were enhanced in beech trees on low-P soil of the Tut forest. In spring, mobilization of P from storage pools in the bark contributed to an effective P cycling. Due to the higher phospholipid “start-up capital” in buds of Tut beeches, the P metabolite profile in developing leaves in spring was similar in beech trees of both forests. During summer, leaves of Tut beeches meet their phosphate (P_i_) needs by replacing phospholipids by galacto- and sulfolipids. Thus, several processes contribute to adequate P_i_ supply on P impoverished soil thereby mediating similar growth of beech at low and sufficient soil-P availability.

## Introduction

Beside nitrogen (N), phosphorus (P) is one of the most important nutrients limiting plant growth and development in terrestrial ecosystems (Lambers et al., 2008, 2010, 2015; Lang et al., 2016). P limitation for terrestrial plants is a consequence of pedogenesis over thousands of years (Lambers et al., 2008; Lang et al., 2016), associated with erosion and leaching processes combined with extremely low atmospheric P deposition (Peñuelas et al., 2013). Some of the most P impoverished soils of the world developed in Australia (Lambers et al., 2012, 2015; Lambers and Plaxton, 2015) and in South Africa at the fynbos biome (Vitousek et al., 2010). Also soils in Central Europe show low P availability as indicated by high foliar N to P ratios of vegetation growing on these soils (Talkner et al., 2009, 2015; Han et al., 2014; Jonard et al., 2015). P deficiency leads to morphological changes such as diminished growth, increased root/shoot ratio and altered root architecture (Lambers et al., 2006; Niu et al., 2013). In addition, physiological changes indicated by modified gene expression and proteome profiles (Lan et al., 2015) are thought to counteract P deficiency. The central consequence of these changes is an efficient phosphorus use in growth and development, photosynthesis, and respiratory energy production (Plaxton and Tran, 2011; Veneklaas et al., 2012; Ellsworth et al., 2015). For example, the replacement of membrane phospholipids by galactolipids, sulfolipids (Lambers et al., 2012), and glucuronosyldiacylglycerol (GlcADG) (Okazaki et al., 2013, 2015) constitutes a strategy providing phosphate (P_i_) to other cellular applications in low P environments and, consequently, improves P_i_ abundance for metabolic processes in plants.

In addition, plants have developed strategies to cope with low P availability in the soil by improving P acquisition and internal P cycling (Côté et al., 2002; Netzer et al., 2017). P acquisition can be improved by (i) root exudation of organic acids and extracellular phosphatases for P solubilization from Al- and Fe-complexes containing P_i_ (Hinsinger et al., 2015; Smith et al., 2015; Tian and Liao, 2015), (ii) increasing P_i_-uptake capacity by enhanced P_i_-transporter expression (Chiou and Lin, 2011), (iii) cluster root formation as found in *Proteaceae* (Lambers et al., 2015), and (iv) mycorrhizal association for optimum soil exploitation (Bucher, 2006; Lambers et al., 2008; Smith et al., 2015). Improved internal P cycling during annual growth includes storage and mobilization of P as well as efficient recycling from leaves before abscission. Both together seem to be a strategy particularly of perennial plants to cope with low P in the environment (Côté et al., 2002; Netzer et al., 2017). This is reminiscent to N nutrition of trees in low-N environments (Rennenberg and Dannenmann, 2015; Sun et al., 2016) and seems a general adaptation strategy to enable high productivity of perennial plants on soil with low nutrient availability *via* relatively closed plant internal nutrient cycles (Rennenberg and Schmidt, 2010; Lang et al., 2016).

In a recent study with adult European beech trees on two field sites in Central Europe with sufficient Conventwald (Con) and low Tuttlingen (Tut) soil-P_i_ availability (Prietzel et al., 2016; Netzer et al., 2017), P (re)cycling was investigated during annual growth. In spring, P_i_ was provided to developing buds and leaves from the storage pools in bark and wood by xylem transport (Netzer et al., 2017) to cover the high amount of P needed for leaf growth and development (Dietz and Foyer, 1986; Plaxton, 1996; Rychter and Rao, 2005; Plaxton and Tran, 2011). Consequently, organic P (P_org_) accumulated in the leaves during summer (Netzer et al., 2017). In autumn, P was re-mobilized from leaves and stored in the bark, wood and dormant buds mostly as P_org_ (Netzer et al., 2017). Similar results were observed for nitrogen with N-storage in the bark (Coleman et al., 1991; Wildhagen et al., 2010), increased occurrence of amino acids (AAs) in the xylem sap during spring (Schneider et al., 1994) and phloem allocation of amino compounds into storage tissues (Schneider et al., 1996; Geßler et al., 1998; Herschbach et al., 2012). These findings led to the hypothesis that also distinct components of the P_org_ fraction must contribute to P storage in stem tissues and to the seasonal dynamics of P_org_ to fulfill the P_i_ demand for metabolic processes needed for growth and development. P_org_ can be attributed to intermediates of photosynthesis, C metabolism, energy generation, and membrane components (Dietz and Foyer, 1986; Plaxton, 1996; Rychter and Rao, 2005; Lambers et al., 2012, 2015) that includes ribosomal RNA, sugar-Ps as well as phospholipids. However, the specific P metabolites that mediate seasonal alterations of the P_org_ fractions in plant tissues at different P_i_ availability in the soil are currently unknown.

The present study was aimed to identify P compounds contributing to the P_org_ pool in leaves, bark, wood, and transport saps as well as to address the specific functions of these metabolites in the seasonal dynamics of P cycling and its dependency on the P_i_ availability in the soil. It was hypothesized that (i) the profile of polar P metabolites and/or phospholipids is modulated by the season in leaves, bark and wood, (ii) independent of tree internal P cycling, the replacement of phospholipids by galacto- and sulfolipids contributes to maintain adequate P_i_ abundance in twig tissues at low-soil-P availability and, (iii) changes in the polar P metabolite and phospholipid profile by both season and P_i_ availability in the soil are associated with changes in central metabolic pathways. To test these hypotheses, polar metabolite and lipid profiles were analyzed in buds/leaves, bark, and wood during annual growth of beech. To understand the connection between metabolic reprogramming of polar P metabolite and phospholipid profiles with storage and mobilization processes, the abundance of P in long-distance transport fluids of xylem and phloem was investigated during spring growth and leaf senescence.

## Materials and methods

### Study sites and plant material

Effects of plant available P_i_ in the soil on the polar metabolome and lipidome were investigated in buds/leaves, bark, and wood, as well as in xylem sap and phloem exudates of adult beech (*Fagus sylvatica* L.) trees. Two forest sites were compared which differ by a factor of eight in soil-P_i_ availability (Netzer et al., 2017). The beech forest site “Conventwald” (Con) represents a low but sufficiently P_i_ supplied forest, whereas “Tuttlingen” (Tut) represents an extremely low-soil-P_i_ forest due to different parent material (Prietzel et al., 2016; Netzer et al., 2017). Both forests are described as mature beech forests and the soils developed on Gneiss containing 0.29 mg P g^−1^ (Con) and on Limestone (Jurassic) (Tut) containing 0.37 mg P g^−1^, respectively (Prietzel et al., 2016). Total P stock of the soil consist of different P species and amounted 162 g m^−2^ for the Con forest and 117 g m^−2^ for the Tut forest (Prietzel et al., 2016). P in the soil of the Tut forest mostly occurred as Ca-bound organic P (pH 6.4–7.4; Prietzel et al., 2016). The adult beech trees of the managed forests originated from natural regeneration and were 160–190 years old at the Con forests (von Wilpert et al., 1996) and 80–90 years old at the Tut forest (Gessler et al., 2001; Pena et al., 2010). Twig tissues from five beech trees were harvested in October 2013, February 2014, April 2014 at bud burst and in June 2014. Xylem sap and phloem exudate were collected in autumn (October 2013) and spring (April 2014). Leaves (or buds in February and bursting buds in April), bark, wood, xylem sap, and phloem exudates were collected from ~30 cm long sun exposed twigs of the beech crown (~25–30 m above ground; Netzer et al., 2017). Xylem sap was collected by the method of Scholander et al. (1965) as modified by Rennenberg et al. (1996). For this purpose, at the cut end the bark of twigs was removed to uncover the wood. The wood was rinsed with *dd*H_2_0 to avoid contaminations and dried with paper tissue. Then, the twig was inserted in a sealed pressure chamber with the cut end protruding. The pressure in the chamber was slowly raised until shoot water potential was reached and kept constant slightly above the shoot water potential to collect xylem sap. The first appearing drops of xylem sap were discarded. The subsequently outrunning xylem sap was collected and frozen in liquid N_2_ until further analyses.

Phloem exudation was performed with bark pieces of ~60 mg on ice in the presence of PVPP (2:1, PVPP/ bark fresh weight) in 10 mM EDTA solution adjusted to pH 7.0 and supplemented with dithiothreitol and the antibiotic chloramphenicol (final concentrations 3 and 0.015 mM, respectively; Rennenberg et al., 1996). Phloem exudation was completed and terminated after 5 h of incubation (Schneider et al., 1996). Samples were shock frozen in liquid N_2_ and stored at −80°C until further processing. Leaf and bark samples were homogenized under liquid N_2_ using mortar and pestle, whereas wood samples were ground under liquid N_2_ using a CryoMill (Retsch, Haan, Germany). All samples were freeze dried at −50°C, at a vacuum of 0.03 mbar for 72 h using the freeze drier Alpha 2–4 (Christ, Osterode am Harz; Germany).

### Lipidome analysis

Lipids in powdered, freeze-dried bud/leaf, bark and wood samples (from five trees at each time point) were extracted and analyzed as described (Okazaki et al., 2015). In total, 28 lipid classes including five P containing lipid classes were identified, i.e., lysophosphatidylcholines (lysoPC, 5 species), phosphatidylcholines (PC, 14 species), phosphatidylethanolamines (PE, 8 species), phosphatidylglycerols (PG, 4 species), and phosphatidylinositols (PI, 2 species). Besides, diacylglycerols (DAG, 8 species) and triacylglycerol (TAG, 39 species), the non-phospholipids sulfoquinovosyldiacylglycerols (SQDG, 7 species), glucuronosyldiacylglycerols (GlcADG, 5 species), glucosylceramides (GlcCer, 7 species), monogalactosyldiacylglycerols (MGDG, 9 species) known to be involved in phospholipid replacement upon P starvation in herbaceous plants (Lambers et al., 2012, 2015; Okazaki et al., 2013, 2015; Siebers et al., 2015) plus digalactosyldiacylglycerols (DGDG, 11 species), acyl steryl glucoside (ASG, 3 species), and sterol glucosides (SG, 3 species) were detected. In each lipid class, lipid species that differ in fatty acid composition, i.e., in total carbon length as well as in double bound abundance, were combined, presented and subjected to statistical analyses.

### Polar metabolome analysis

The powdered and freeze-dried tissue samples as well as freeze dried xylem sap samples and phloem exudates (3 replicates per harvest, each) were analyzed by CE-MS according to Oikawa et al. (2011a,b).

### IRMS measurements

Three milligrams of dried, fine milled, and homogenized plant material was weight into tin boats using a precision scale. The samples were combusted in an elemental analyzer (Euro EA 3000, Hekatech, Wegberg, Germany) and analyzed in a continuous-flow isotope ratio mass spectrometer (IsoPrime, Elementar, Stockpor, UK) (Werner et al., 2006). The samples were measured against reference standard IAEA-600 caffeine and IAEA-NO-3 potassium nitrate (repeated measurement precision was 0.10) for nitrogen, and IAEA-600 caffeine and IAEA-CH-3 cellulose (repeated measurement precision was 0.12) for carbon.

### Statistical analyses

To characterize seasonal fluctuations and differences between field sites, polar metabolome, and lipidome data were subjected to partial least square discriminant analysis (PLS-DA) using MetaboAnalyst 3.0 (Xia et al., 2015). To reach maximum separation in PLS-DA plots, data were normalized by median, log transformed, and auto scaled (mean centered and divided by the standard deviation of each variable). Heat maps were created using the raw data subjected to Pearson's correlations analyses. As similarity measure Ward's clustering algorithm was used. Significant seasonal fluctuations of individual lipids (*n* = 5) or metabolites (*n* = 3) within one tissue and forest site were analyzed with One Way ANOVA (Holm–Sidak test as *post-hoc* test) in case of normal distributed data. In case of not normal distributed data or if data showed inhomogeneity of variances, the Kruskal–Wallis ANOVA test was applied (both *p* ≤ 0.05, α = 0.95). Normal distribution of the data was tested with the Shapiro–Wilk test; the homogeneity of variances was tested with the Levene^2^ test (both *p* ≤ 0.05). Statistically significant differences of individual lipids or polar metabolites within each season between study sites were analyzed by Student's *t-*test in case of normal distributed data. In the case of not normal distributed data or if homogeneity of variances was not given, the Mann–Whitney test was performed. Interactions between lipids and polar metabolites were analyzed by calculating Pearson's correlation coefficients. Statistical analyses were conducted with the Origin PRO 9.1 software (OriginLab Corporation, Northampton, USA). Venn diagrams were used to identify polar metabolites and lipids of tissues, which contribute to the separation of seasons and sites in each twig tissue (http://bioinformatics.psb.ugent.be/webtools/Venn/).

## Results

### Total C, N, and P in twig leaves/tissues changed during annual growth

**Figure 1:**
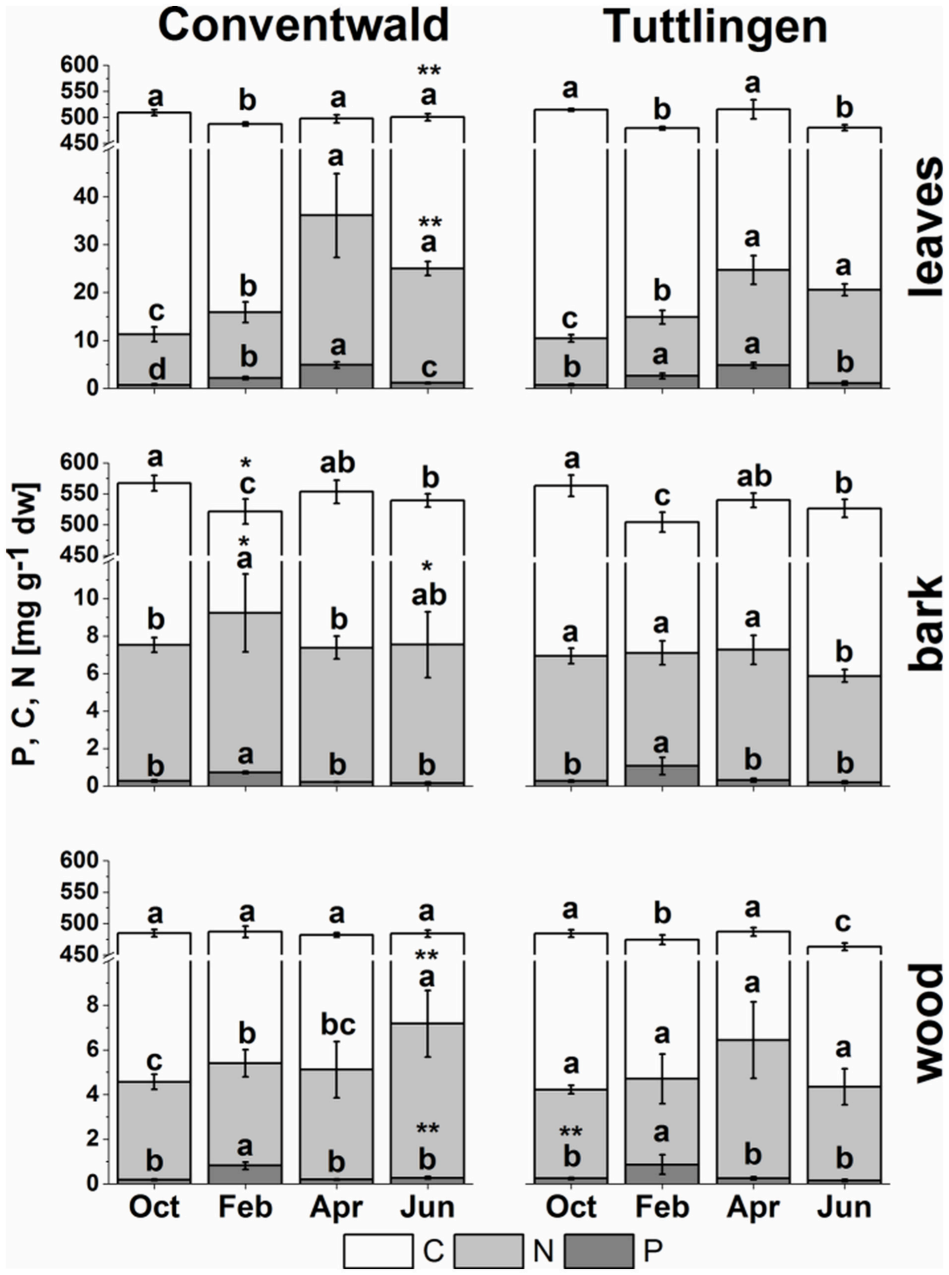
Total C, N, and P in buds/leaves, bark, and wood of adult beech trees. The top of the bars represents mean values ± *S.D*. of total C (white bars), total N (light gray bars) and total P (dark gray bars) in beech twig organs/tissues. Data of total P were taken from Netzer et al. (2017). Lower case letters represent significant differences between seasons determined by Mann–Whitney-tests after Kruskal–Wallis-ANOVA as global test at the *p* ≤ 0.05 level per tissue and site. Asterisks indicate significantly higher C, N, or P contents in the respective organ/tissue compared to the other field site. ^*^p ≤ 0.05; ^**^p ≤ 0.01 (results of Student's *t-*test or Mann–Whitney tests in case that the requirement of normal distribution of the data was not fulfilled). (Oct, October; Feb, February; Apr, April; Jun, June).

Total C, N (present study), and P (Netzer et al., 2017) analyses revealed clear seasonal changes indicating P and N storage in bark and wood during dormancy and its mobilization during spring (Figure 1). In leaves, C was highest in senescent leaves and lowest in bursting buds. By contrast, N and P in leaves were lowest in autumn and highest in spring (Figure 1, Netzer et al., 2017). This pattern was observed in leaves of both forest sites, whereas the N increase in spring was less pronounced in leaves from the low-P and low-N forest site Tut. In leaves, P and N declined from summer until autumn indicating mobilization before leaf abscission. Bark and wood showed storage of P during dormancy at both field sites, but storage of N only in Con beeches (Figure 1, Netzer et al., 2017). Irrespective of seasonal changes, N in bark and wood was lower in Tut compared to Con beech trees in early summer (Figure 1). Surprisingly, the C content in the bark of both, Tut and Con beech trees showed lowest values during winter when C storage was expected. Analyses of the polar (P) metabolome and (P) lipidome were performed to identify distinct P and N compounds contributing to the seasonal fluctuations and site specific differences of P, N, and C in twig tissues.

### The contribution of P metabolites to the polar metabolome and lipidome of beech twigs

The polar metabolome and the polar P metabolome profile were first characterized by Venn analyses to get insight into differences between forests sites, twig organs/tissues and transport fluids (Supplementary Figure S1, Supplementary Table S1). While the overall number of polar (P) metabolites was equal in buds/leaves, bark, wood, xylem sap, and phloem exudates at both study sites, Venn-analyzes elucidated five leaf-specific, two xylem sap-specific [(G1P; Gal1P) and dAMP] and one phloem exudate specific (NaMN) polar P metabolite (Table 1). The lipidome of buds/leaves and wood showed 28 lipid classes, while 27 lipid classes were found in the bark. In leaves/buds, bark and wood all five classes of phospholipids (lysoPC, PC, PE, PG, PI) were found. Besides DAG and TAG, the non-phospholipids SQDG, GlcADG, GlcCer, and MGDG, all known to be involved in phospholipid replacement upon P starvation in herbaceous plants (Okazaki et al., 2013, 2015; Siebers et al., 2015), plus DGDG, ASG, and SG were detected (Supplementary Table S2).

**Table 1:**
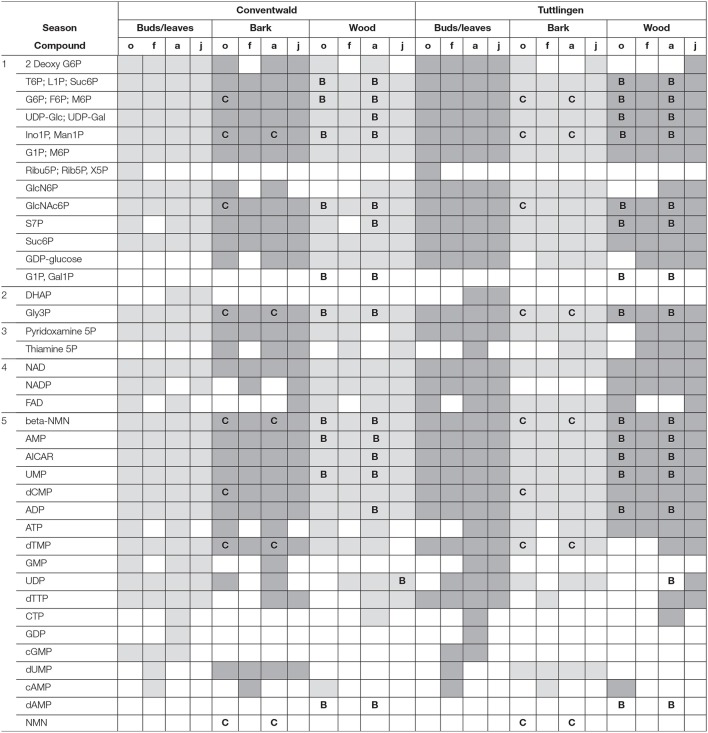
Organ/Tissue specific abundances of P metabolites.

*P metabolites in buds/leaves, bark and wood of Fagus sylvatica in October (o), February (f), April (a), and June (j) of both forests, Conventwald (Con) and Tuttlingen (Tut). Gray shaded boxes = P metabolite is present in this tissue during this season. White boxes = not detected. 1 = sugar phosphates, 2 = triose phosphates, 3 = coenzymes, 4 = dinucleotides, 5 = nucleotides. B = present in the xylem sap. C = present in phloem exudates at this study site and during this season. The complete list of all metabolites is given in Supplementary Table S1*.

Partial Least Square Distance Analyses (PLS-DA) identified seasonal differences in the lipidome and polar metabolome of beech twig leaves/tissues and revealed its dependency on P_i_ availability in the soil (Supplementary Figure S2). The lipidome and the polar metabolome of buds/leaves was different for all seasons and between levels of P_i_ availability in the soil. Phospholipids contributed 19–20%, P metabolites 13% to the differentiation of the metabolome and lipidome, respectively (Supplementary Tables S3, S4). The polar metabolome of bark and wood varied between seasons at both study sites, but with a lower contribution of P metabolites of 13% (Con) and 10% (Tut), whereas the lipidome of bark and wood showed only minor seasonal differences. The xylem sap and phloem exudate polar metabolome showed clear differences between spring and autumn as well as for both study sites (Supplementary Figure S3). The contribution of polar P metabolites to the metabolome variation was below 10%.

Differences in the polar metabolome and lipidome profile were also observed between both forest sites. The lipidome of leaves/buds was different in spring and autumn, while the polar metabolome was divergent in summer and autumn. The wood lipidome varied between sites in summer and autumn, while the polar metabolome of the wood differed between sites in spring, summer and autumn. VIP-scores of the PLS-DA analyses showed that the differentiation of the polar metabolome by season and site was mainly mediated by N containing metabolites rather than P metabolites (Supplementary Table S3). Several phospholipids were among the top 10 lipids contributing to the differentiation in PLS-DA plots (Supplementary Tables S3, S4). This clearly indicates phospholipids and N compounds as drivers to distinguish seasons and sites. Identification of distinct N compound(s) and phospholipids that differ between seasons and sites are elucidated below.

### Polar P metabolites and phospholipids responsible for seasonal fluctuations in total P and N

#### Remodeling of the polar metabolome and lipidome in beech twigs until autumn and winter indicates mobilization and storage

A special feature of the perennial life style of deciduous trees such as European beech is the remobilization of nutrients from senescent leaves for storage in bark and wood in autumn (Millard and Grelet, 2010; Rennenberg and Schmidt, 2010; Rennenberg et al., 2010). In the present study, decreased levels of chlorophyll and carotenoids in autumn leaves indicated leaf senescence (Supplementary Figure S4) and thus preparation of the adult beech trees for dormancy.

##### Leaves

**Figure 2:**
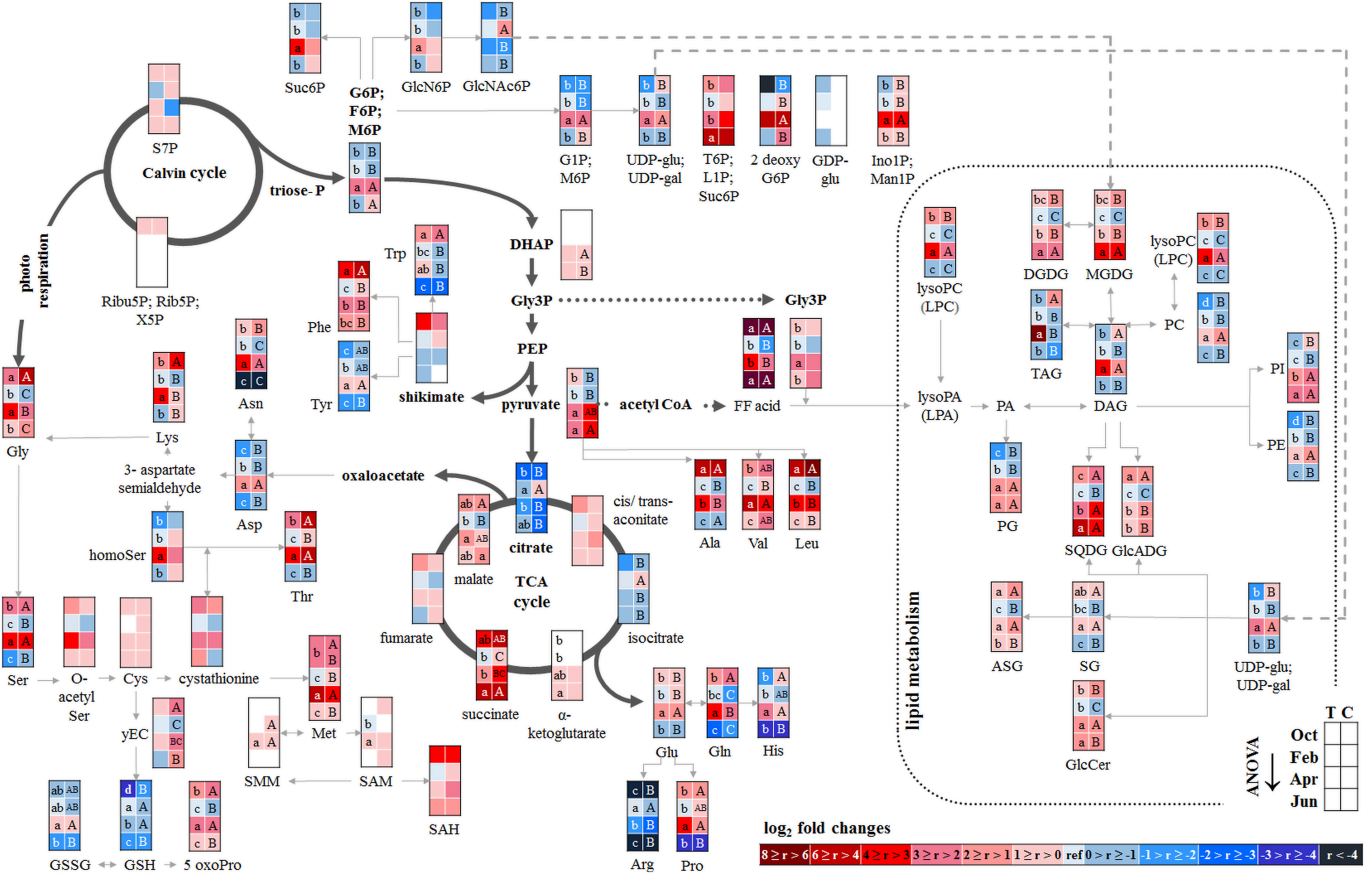
Changes in polar metabolite and lipid abundances during annual growth in leaf buds and leaves. The figure shows heat maps of sugar-Ps, intermediates of central carbon metabolic pathways, and amino acids including sulfur compounds and lipids in senescent leaves (October) dormant leaf buds (February), bursting buds/developing leaves (April), and mature leaves (June) of adult beech trees from the Tut (T) and Con (C) forest. Mean values of seasonal fluctuations of metabolites (*n* = 3) and lipids (*n* = 5) were presented as log_2_ fold changes using Tut data in February as reference. Different minor (Tut) or capital (Con) letters indicate significantly different relative metabolite or lipid abundance between the four seasons per site (results of One-Way-ANOVA, or Kruskal–Wallis-ANOVA in case that the requirement of normal distribution and/or equal variances failed; *p* < 0.05). White = not detected. Abbreviations of metabolites: γ EC, γ-glutamylcysteine; GlcNAc6P, N-acetyl-D-glucosamine-6-phosphate; GlcN6P, glucosamine-6-phosphate; OPH, O-phospho-L-homoserine; SAH, S-adenosyl-L-homocysteine; SAM, S-adenosyl-L-methionine. Abbreviations of lipids: ASG, acyl steryl glucoside; DAG, diacylglycerol; DGDG, digalactosyldiacylglycerol; FF acid, free fatty acid; GlcADG, glucuronosyldiacylglycerol; GlcCer, glucosylceramide; Gly3P, glycerol-3-phosphate; lysoPA, lysophosphatidic acid; lysoPC, lysophosphatidylcholine; MGDG, monogalactosyldiacylglycerol; PA, phosphatidic acid; PC, phosphatidylcholine; PE, phosphatidylethanolamine; PG, phosphatidylglycerol; PI, phosphatidylinositol; SG, steryl ester; SQDG, sulfoquinovosyldiacylglycerol; TAG, triacylglycerol. Abbreviations of seasons: Oct, October; Feb, February; Apr, April; Jun, June. The pathways were designed based on Kegg pathways (http://www.genome.jp/kegg/pathway.html?sess=2764b8338258d6286de91bbebe6faf46) and on Hirabayashi and Ichikawa (2002), Gardocki et al. (2005), Li-Beisson et al. (2010), Boudière et al. (2014), Guschina et al. (2014), Okazaki et al. (2013, 2015), Furo et al. (2015), Kobayashi (2016), and Galili et al. (2016).

P resorption from senescent leaves can occur either directly as P_org_ or as P_i_ after cleavage from organic P compounds. The latter was indicated by lower abundance of phospholipids (PE, PG, PC, and PI) in autumn compared to summer in senescent beech leaves from Tut, and for PG and PI in senescent beech leaves from Con beech trees (Figure 2). The simultaneous increase in the abundance of lysoPC, an indicator of phospholipid degradation (Nakamura, 2013; Boudière et al., 2014), supports this assumption (Figure 2).

**Figure 3:**
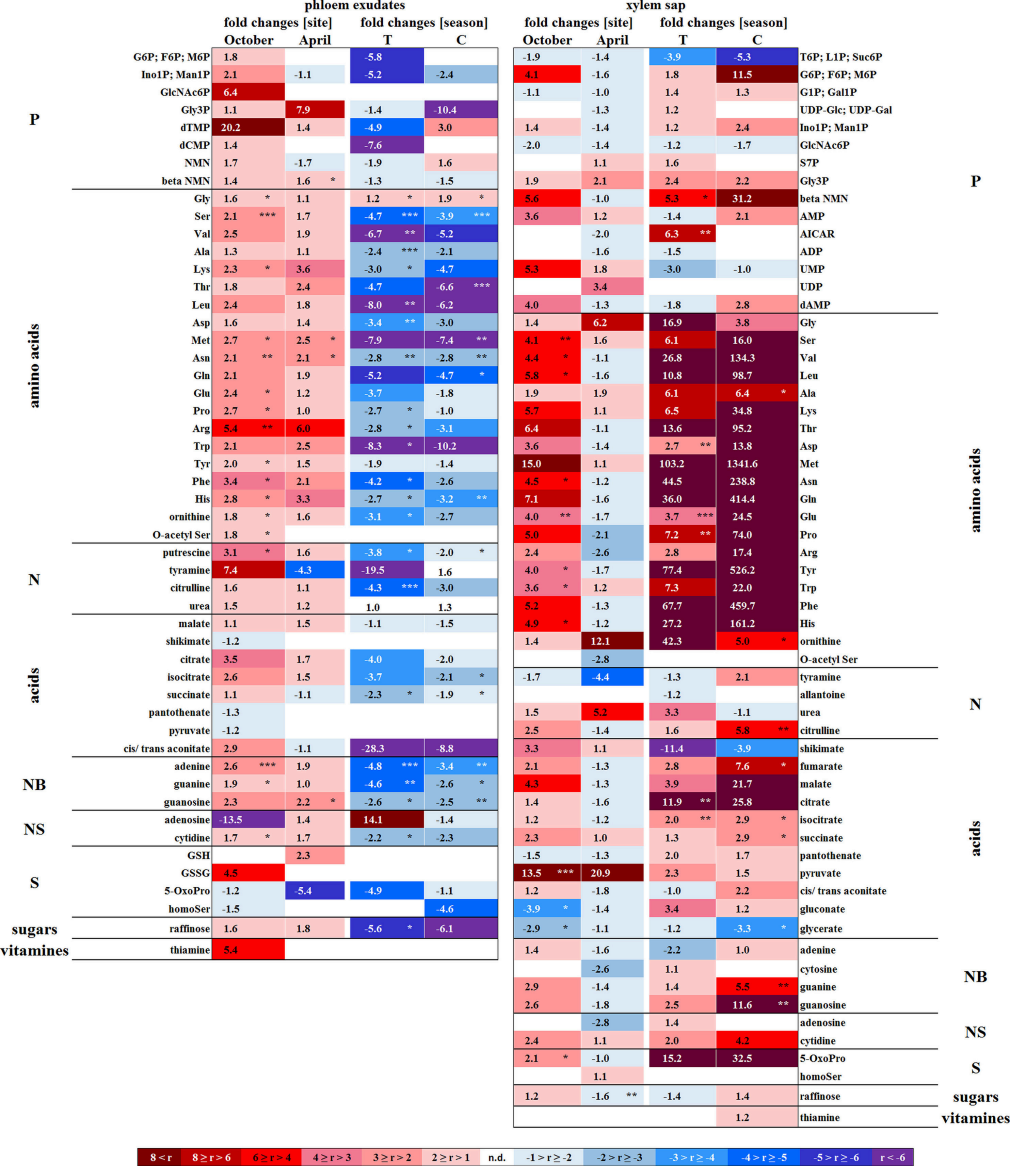
Site and season effects on the polar metabolite abundance in xylem saps and phloem exudates. Phloem exudates (left) and xylem saps (right) from twigs of adult beech trees of the Tut (T) and Con (C) forest in autumn (October) and spring (April) are shown. Mean values (*n* = 3) of fold changes of relative peak areas are presented. The higher metabolite abundance in xylem saps and phloem exudates of Tut beeches at both seasons is indicated as Tut/Con ratios (red), while the lower abundance of metabolites in Tut samples is indicated by the reciprocal Con/Tut ratio (blue). Seasonal differences are presented as ratios of fold changes of relative peak values between April/October. Asterisks indicate significant differences for a particular metabolite between both field sites and between both seasons (results of Student's *t-*test or Mann–Whitney tests in case that the requirement of normal distribution of the data was not fulfilled). ^*^p < 0.05; ^**^p < 0.01; ^***^p < 0.001. nd = not detectable. Relative peak areas of all identified metabolites are given in the Supplementary Table S1.

In senescent leaves, the levels of most sugar-Ps remained unaffected compared to beech leaves in summer (Figure 2); products of glycolysis were either not detectable (DHAP) or lower (pyruvate) in autumn compared to summer. N remobilisation from senescent leaves after protein breakdown was indicated by the accumulation of both, numerous AAs (Figure 2) and the coenzyme pyridoxamine5P involved in the amino acid metabolism in autumn (Guo et al., 2004; Roje, 2007). In addition, the abundances of glutathione (GSH), the predominant transport form of reduced sulfur in the phloem of beech (Herschbach and Rennenberg, 1996), and 5-oxoproline, a degradation product of GSH (Bergmann and Rennenberg, 1993), in leaves were higher in autumn compared to summer. Accumulation of AAs, including sulfur (S) containing compounds, in phloem exudates in autumn compared to spring (Figure 3) coincide with their allocation to the storage tissues bark and wood via the phloem. Furthermore, raffinose, an important phloem mobile sugar (Rennie and Turgeon, 2009; Turgeon and Wolf, 2009), was ~5-fold higher in phloem exudates in autumn compared to spring.

##### Bark

Changes in polar metabolite abundances that indicate nutrient storage in bark parenchyma cells during autumn were mostly driven by AAs (Supplementary Table S3). However, only the abundance of Val, Leu, and Gln was higher in autumn than in summer but significantly declined until dormancy. In case of amino-N storage in form of storage proteins, as previously reported for poplar (Wildhagen et al., 2010), this is not surprising and fits well with the higher total-N (Figure 1) content in the bark of Con beeches during dormancy.

**Figure 4:**
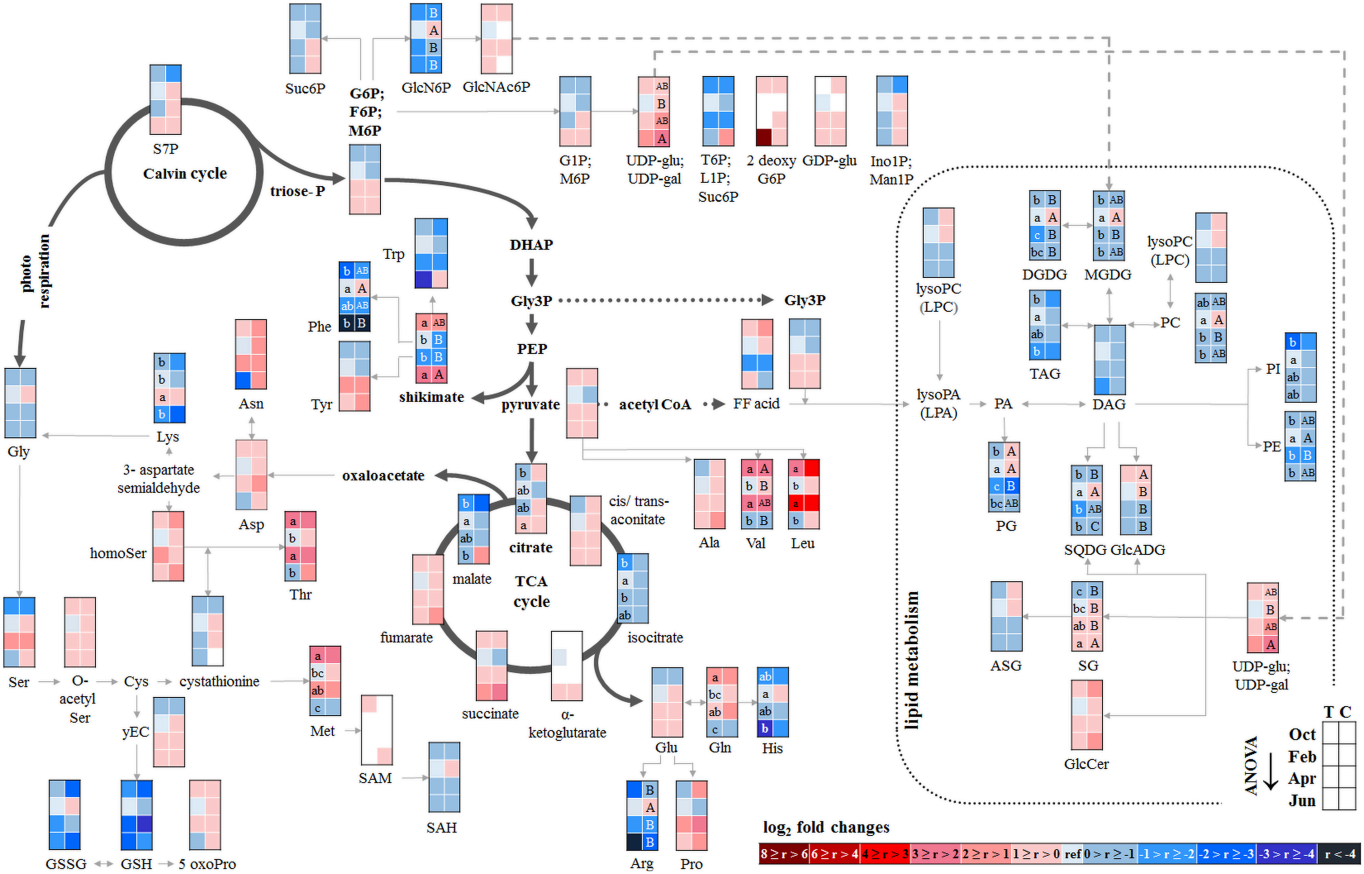
Changes in polar metabolite and lipid abundances during annual growth in the bark. The figure shows heat maps of sugar-Ps, intermediates of central carbon metabolic pathways, and amino acids including sulfur compounds and lipids in the twig-bark of adult beech trees from the Tut (T) and Con (C) forest. Mean values of seasonal fluctuations of metabolites (*n* = 3) and lipids (*n* = 5) were presented as log_2_ fold changes using Tut data in February as reference. Different minor (Tut) or capital (Con) letters indicate significantly different relative metabolite or lipid abundance between the four seasons per site (results of One-Way-ANOVA, or Kruskal–Wallis-ANOVA in case that the requirement of normal distribution and/or equal variances was not fulfilled; *p* < 0.05). White = not detected. Abbreviations see Figure 2.

In contrast, total-C in the bark was higher in autumn than in summer and was lowest during dormancy (Figure 1). Fat in oleosomes can constitute a C storage pool in the bark of deciduous trees (Sauter and van Cleve, 1994) and increased lipid levels can be expected in autumn and/or later during dormancy. Against this assumption, lipids, including phospholipids in the bark during autumn remained similar to summer levels (Figure 4). Nevertheless, the storage lipid TAG (Xu and Shanklin, 2016) and the phospholipids PC, PE and PG increased from autumn until winter. This effect was significant only for the bark of Tut beeches (Figure 4) and, this increase coincided with highest P_tot_ levels in the bark during dormancy (Figure 1). In addition, the abundances of MGDG, DGDG, SQDG, and of glucosamine-6-phosphate (GlcN6P, significant for Con beech twigs), one precursor of glycolipid synthesis (Furo et al., 2015), were higher in the twig-bark during dormancy compared to autumn.

##### Wood

**Figure 5:**
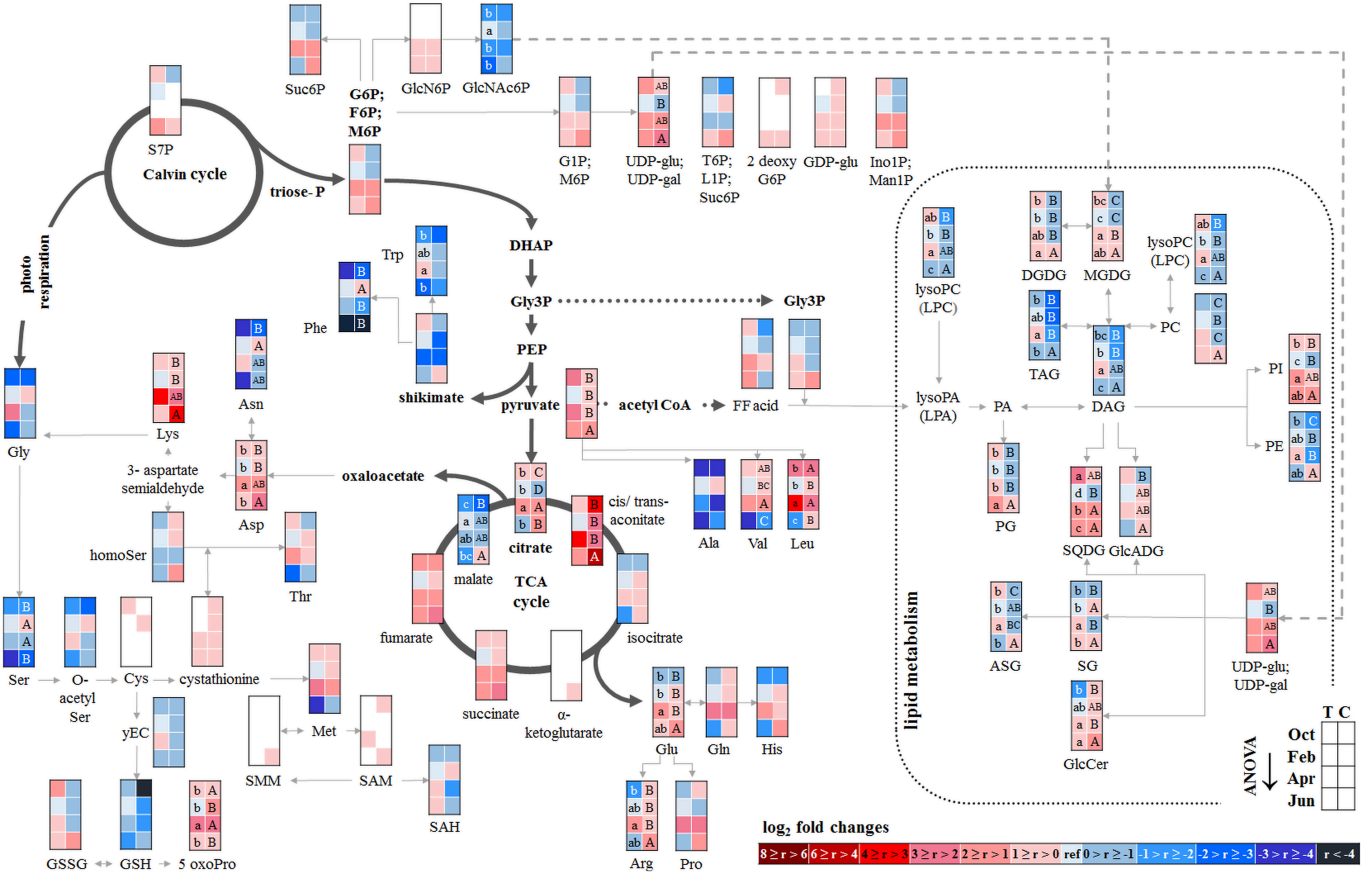
Changes in polar metabolite and lipid abundances during annual growth in the wood. The figure shows heat maps of sugar-Ps, intermediates of central carbon metabolic pathways, and amino acids including sulfur compounds and lipids in the twig-wood of adult beech trees from the Tut (T) and Con (C) forest. Mean values of seasonal fluctuations of metabolites (*n* = 3) and lipids (*n* = 5) were presented as log_2_ fold changes using Tut data in February as reference. Different minor (Tut) or capital (Con) letters indicate significantly different relative metabolite or lipid abundance between the four seasons per site (results of One-Way-ANOVA, or Kruskal–Wallis-ANOVA in case that the requirement of normal distribution and/or equal variances was not fulfilled; *p* < 0.05). White = not detected. Abbreviations see Figure 2.

The twig-wood revealed only minor changes in polar metabolite abundances between summer and autumn (Figure 5). Levels of sugar-Ps remained unaffected and the abundances of organic acids involved in glycolysis and the TCA-cycle, declined only in the wood of Con beeches. Significant changes were observed within the lipidome (Figure 5). The abundances of the phospholipids PG (both sites), PC, and PE (Con only) declined from summer to autumn, as also observed for the galactolipids DGDG and MGDG, while SQDG increased (Tut only). Later during dormancy, the abundance of PC, lysoPC and PE increased in the Con twig-wood, when P_i_ declined (Netzer et al., 2017). This indicates the use of P_i_ for phospholipid formation and phospholipid storage during dormancy. Furthermore, the P containing hexose N-acetyl-D-glucosamine 6-phosphate (GlcNAc6P), was highest in twig-wood and may contribute to P storage during dormancy as indicated by highest P_tot_ levels (Figure 1).

#### Remodeling of the polar metabolome and lipidome in spring indicates mobilization of twig storage pools

In spring, growth of deciduous trees is characterized by the development of new leaves from buds and by stimulation of metabolic activity. Accordingly, the abundances of chlorophyll and carotenoids in buds increased compared to dormancy (Supplementary Figure S4), whereas ABA that keeps buds dormant in winter (Fladung et al., 1997) disappeared (Supplementary Figure S5). In the xylem sap lowered ABA, but higher levels of tZ (trans-zeatin), GA3 and GABA were observed in spring compared to autumn (Supplementary Figure S9) and correspond with their individual functions in dormancy release (Fladung et al., 1997; Zheng et al., 2015).

##### Developing buds

In developing buds, sugar-Ps and triose-Ps as products of hexose degradation by glycolysis, such as DHAP (only detectable in spring and summer) and pyruvate (Supplementary Figure S6), were higher in spring compared to dormancy. Simultaneously, AAs synthesized from phosphoenolpyruvate (Tyr, Phe, Try) and pyruvate (Ala, Leu, Val) also increased. The higher level of pyruvate coincides with enhanced malate and succinate levels in beech buds although citrate decreased (Figure 2). This fits well with increasing metabolic activities expected in spring during bud swelling and bud break. In accordance, AAs synthesized from TCA-cycle intermediates, i.e., 2-oxoglutarate (Glu-family) and oxaloacetate (Asp-family), peaked in spring (Figure 2). Although net photosynthesis is low in developing buds (Umeki et al., 2010), the AAs build from 2-phosphoglycolate via photorespiration, i.e., Ser and Gly, increased. Both AAs may also be synthesized via metabolic pathways different from photorespiration (Ho and Saito, 2001; Benstein et al., 2013) and/or may be supplied *via* xylem transport. Indeed, comparison of the xylem sap polar metabolome in spring with autumn revealed higher abundances of several organic acids, proteinogenic AAs including Ser and Gly, and several compounds of the TCA-cycle, namely citrate, isocitrate, succinate, fumarate, and malate, which can all be transported into the developing buds in spring.

In bursting buds, degradation of organic P compounds such as phospholipids to supply P_i_ for energy metabolism was not indicated. In opposite, in bursting buds, the abundances of all phospholipids, i.e., PC, PE, PG, PI, and lysoPC increased instead of decreased from dormancy until spring (Figure 2). Apparently, phospholipids in buds constitute sinks rather than sources for P_i_ at the beginning of the vegetation period. Another possible P_i_ source may be GlcNAc6P that is also a precursor of MGDG and DGDG synthesis (Supplementary Figure S8). GlcNAc6P declined in buds until spring although its precursor GlcN6P increased (Figure 2). This corresponds furthermore with high (UDP-Glc; UDP-Gal) levels in bursting buds. Hence, the present results indicate synthesis of non-phospholipid rather than phospholipid degradation.

##### Bark and wood

The polar metabolite and lipidome profile in both, bark and wood was mostly similar between dormancy and spring. Among sugar-Ps or organic acids involved in glycolysis and the TCA-cycle, only succinate in the bark and citrate in the wood increased in spring compared to dormancy (Figure 4). In the twig-wood, lipids were not involved in C-mobilization during spring, because MGDG (both field sites) as well as the storage fats TAG and DAG (Tut wood only) increased compared to dormancy (Figure 5). In contrast, protein breakdown in bark and wood during spring is indicated by enhanced abundances of AAs in these tissues (Figures 4, 5). These AAs may be loaded into the xylem as denoted by the generally higher amino acid abundances in the xylem sap during spring (Figure 3).

In the xylem sap of adult beech trees also P_i_ strongly increased in spring (Netzer et al., 2017). GlcN6P in the bark and GlcNAc6P in the wood declined until bud burst and may constitute a source of xylem transported P_i_. Further candidates providing P_i_ were the phospholipids PC, PG, and PE that declined in the bark during spring compared to dormancy. In contrast, phospholipids in the wood did not change between dormancy and spring or even increased and, thus, did not provide P_i_ for the xylem transport. beta-NMN that also increased in the bark, wood (Supplementary Figure S10) and xylem sap (Figure 3) during spring seems to be a candidate contributing to the enhanced P_org_ pool in the xylem sap at this time of the year.

### Soil-P and soil-N influence the P/N metabolome and phospholipid profile

#### The polar metabolome and the lipidome in beech twigs during autumn and dormancy depend on the P_i_ availability in the soil

Both, the low plant available soil-P_i_ (Netzer et al., 2017) and soil-N (Rennenberg and Dannenmann, 2015) at the Tut site caused particular differences in the polar metabolome and lipidome profile in beech twig organs/tissues from Tut compared to Con beech trees in autumn and dormancy.

##### Senescent leaves

**Figure 6:**
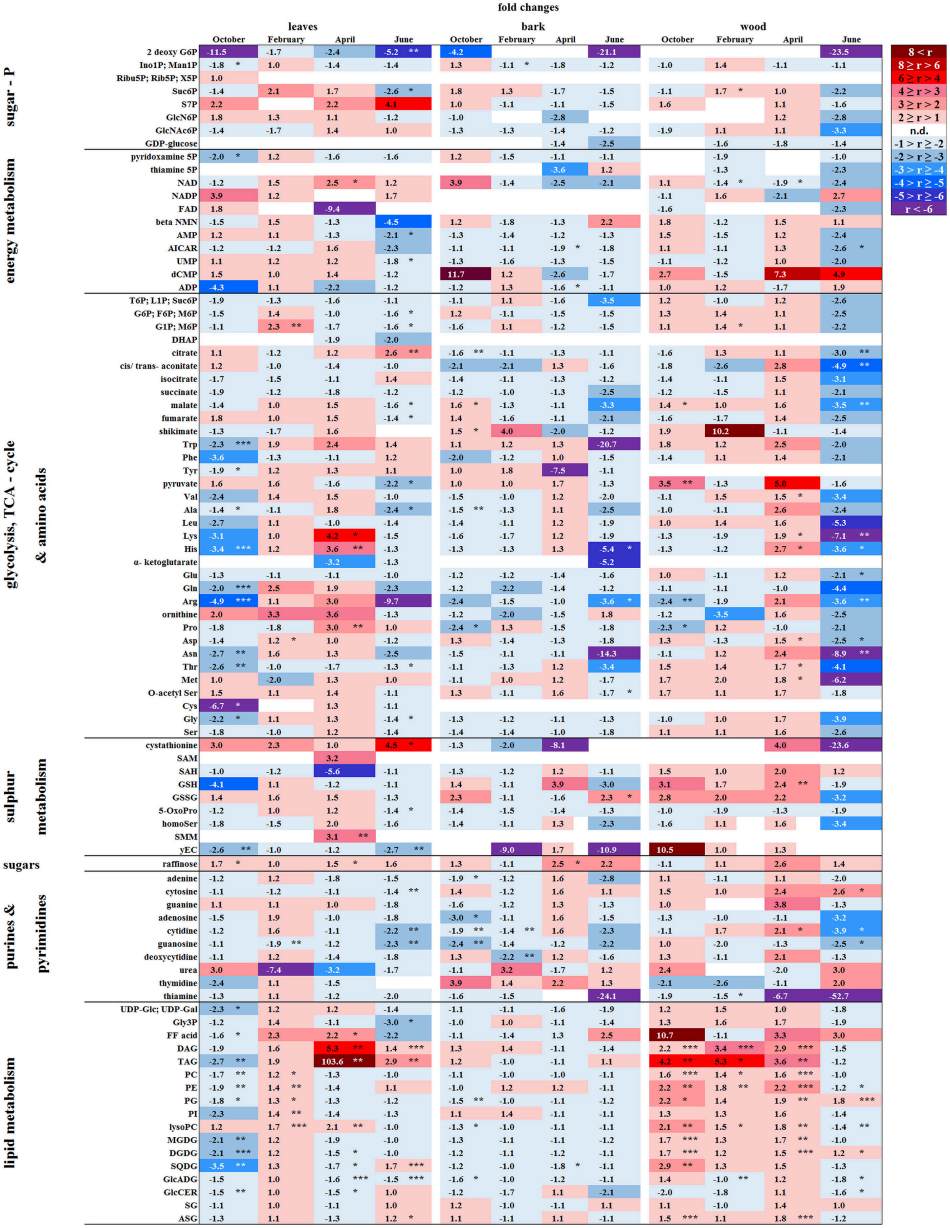
Alterations in polar metabolite and lipid abundance due to P limitation. Fold changes of polar metabolites (*n* = 3) and lipids (*n* = 5) in buds/leaves, bark and wood of beech twigs from adult beeches of the Tut (low soil-P) and Con (sufficient soil-P) forests. Mean values of fold changes in relative peak areas are presented for the different seasons, i.e., autumn: October, dormancy: February, spring: April and early summer: June. The higher metabolite abundance in Tut tissues is given by Tut/Con ratios (red). The lower abundance of metabolites in Tut samples is presented by reciprocal Con/Tut ratios (blue). Asterisks indicate significant differences within one metabolite between both field sites during one season (results of Student's *t*-test or Mann–Whitney test in case that the requirement of normal distribution of the data was not fulfilled). ^*^p < 0.05; ^**^p < 0.01; ^***^p < 0.001; white boxes = not detectable. Relative peak areas of all identified metabolites are given in the Supplementary Table S1. AICAR, 5-aminoimidazole-4-carboxamide ribonucleotide; ASG, acyl steryl glucoside; DAG, diacylglycerol; DGDG, digalactosyldiacylglycerol; DHAP, dihydroxyacetone phosphate; FF acid, free fatty acid; γEC, γ-glutamylcysteine; GlcADG, glucuronosyldiacylglycerol; GlcCer, glucosylceramide; GlcN6P, glucosamine-6-phosphate; GlcNAc6P, N-acetyl-D-glucosamine-6-phosphate; Gly3P, glycerol-3-phosphate; lysoPA, lysophosphatidic acid; lysoPC, lysophosphatidylcholine; MGDG, monogalactosyldiacylglycerol; OPH, O-phospho-L-homoserine; PA, phosphatidic acid; PC, phosphatidylcholine; PE, phosphatidylethanolamine; PG, phosphatidylglycerol; PI, phosphatidylinositol; SAH, S-adenosyl-L-homocysteine; SAM, S-adenosyl-L-methionine; SG, steryl ester; SQDG, sulfoquinovosyldiacylglycerol; TAG, triacylglycerol.

Except for two sugar-Ps, (Ino1P; Man1P) and 2deoxyG6P, that were significantly lower in senescent Tut leaves (Figure 6), polar metabolites of central metabolic pathways were indifferent between both forest sites. By contrast, numerous AAs were significantly lower in senescent leaves from the low-soil-P_i_ (and N) forest Tut than in senescent leaves from Con beeches (Figure 6). This finding was accompanied by the higher abundance of numerous AAs, some nucleobases and nucleosides in phloem exudates of Tut beeches (Figure 3) and indicates enhanced phloem transport of mobilized N from Tut beech leaves. A similar result was obtained for P_i_ mobilization from phospholipids such as PC, PE, and PG, which were less abundant in senescent leaves of Tut compared to Con beech trees (Figure 6). Furthermore, the non-phospholipids MGDG, DGDG, and SQDG as well as glucosylceramide (GlcCer) and free fatty (FF) acids were lower in senescent leaves from Tut than from Con beech trees, whereas similar levels were found for DGDG and MGDG in summer. The lower levels of these constituents of chloroplast membranes (Kobayashi, 2016) correlated with lower chlorophyll and carotenoid contents in senescent leaves from Tut compared to Con leaves (Supplementary Figure S4). All these findings indicate enhanced nutrient resorption from Tut compared to Con leaves.

##### Dormant buds

In both, dormant Tut and Con beech buds, ABA reached the highest level during the year (Supplementary Figure S5), as expected from the function of this phytohormone in setting and maintaining the dormant state (Fladung et al., 1997; Zheng et al., 2015). Although polar metabolites were not different between dormant Tut and Con buds, all phospholipids (PC, PE, PI, PG, lysoPC) showed higher abundances in dormant buds of Tut than of Con beech trees (Figure 6).

##### Bark and wood

With few exceptions, the polar metabolite profile was similar in the twig-bark and wood of beech trees from both forest sites during autumn (Figure 6). Only minor differences in metabolites involved in glycolysis and the TCA-cycle were observed. Nevertheless, abundances of several AAs were lower in twig-wood and/or bark of Tut beeches (Figure 6). Even the bark lipid profile showed only minor differences between the two forest sites in autumn whereas the wood lipid profile showed strong site-specific differences (Figure 6). Compared to Con beech trees, the abundances of phospholipids were higher in the wood of Tut beech trees during autumn (PC, PE, PG, and lysoPC) and dormancy (PC, PE, and lysoPC). The wood of Tut twigs also showed higher amounts of nearly all non-phospholipids, such as MGDG, DGDG, SQDG, TAG, DAG, ASG, and FF acids in autumn. These differences vanished during subsequent dormancy for MGDG, DGDG, and SQDG but PE, PC, the storage fats TAG, DAG, and FF acids remained higher in the wood of Tut beeches during dormancy (Figure 6). Altogether, this strongly indicates higher investment in P storage in form of phospholipids in the wood of Tut compared to Con beech trees as observed for the buds of Tut beeches (see above).

#### Mobilization of P from twig storage pools in spring is modulated by the P_i_ availability in the soil

##### Bursting buds

The polar metabolome profile of Con and Tut beech buds showed only few differences during spring. At the two forest sites the abundance of phospholipids was comparable, but the levels of TAG, DAG, and FF acid were higher in bursting buds of Tut compared to Con beech trees. Surprisingly, lipids involved in phospholipid replacement such as DGDG, SQDG, GlcADG, and GlcCER (Okazaki et al., 2013, 2015; Siebers et al., 2015) were higher in Con than in Tut buds in spring (Figure 6). Hence, swelling buds did not reveal any indication that low soil-P_i_ influenced their P metabolism. Regardless, the composition of the phloem sap that connects buds as sink tissues with stem tissues as nutrient sources showed few differences between beeches of both forests. During spring phloem exudates of Tut trees revealed higher abundances of beta-NMN, Met, Asn, and guanosine compared to Con beech trees (Figure 3).

##### Bark and wood

In the bark, polar metabolite abundances did not differ between beech trees of both forest sites in spring. In contrast, polar metabolites of the twig-wood revealed significant differences in AA levels. Val, Lys, His, Asp, Thr, and Met plus the sulfur containing peptide glutathione were higher in Tut than in Con twig-wood (Figure 6). Furthermore, the lipidome profile of the wood in spring showed strong differences between both forests as observed during autumn and dormancy. Several phospholipids and non-phospholipids were higher in the twig-wood of Tut compared to Con beech trees (Figure 6). These compounds included the phospholipids PC, PE, PG and lysoPC, the galactolipids DGDG, MGDG, and the storage lipids TAG and DAG (Bates and Browse, 2012; Manan et al., 2017) (Figure 6).

#### The polar metabolome and the lipidome of beech twigs are modulated by the P_i_ availability in the soil during vegetative growth in summer

Despite the lower soil-P_i_ availability at the Tut compared to the Con forest (Prietzel et al., 2016; Netzer et al., 2017), total P in leaves and other tissues was similar in beeches of both forests sites in summer indicating balanced P nutrition (Figure 1; Netzer et al., 2017). Still, distinct differences observed in the polar metabolome and lipidome indicate strategies to maintain adequate P_i_ levels in the leaves of Tut beech trees *via* bypassing the use of P_i_ in C metabolism and *via* phospholipid replacement by galacto- and sulfolipids.

##### Leaves

Leaf photosynthesis produces carbohydrates to supply leaf metabolism, and heterotrophic tissues/organs such as roots, bark and wood with carbohydrates. Since chlorophyll and carotenoid levels were similar in both, Tut and Con beech leaves (Supplementary Figure S4) photosynthetic capacity can be expected to be equal in beech leaves of the two forest sites. Despite this similarity, several P containing hexoses involved in C metabolism, i.e., 2deoxyG6P, (G6P; F6P; M6P), (G1P; M6P), and sucrose-6P (Suc6P), were significantly lower in leaves from Tut compared to Con beech trees (Figure 6). These differences correspond to both, significantly lower C (Figure 1) and P_org_ (Netzer et al., 2017) contents in Tut leaves in summer. The abundance of sedoheptulose-7P (S7P) involved in ribulose-5P regeneration in the Calvin cycle was higher in Tut leaves. This might indicate insufficient P_i_ abundance for sugar-P synthesis via the Calvin cycle. In accordance, the abundance of the amino acids Gly and Ser, synthesized by photorespiration from 2-phosphoglycolate (Supplementary Figure S7), were slightly reduced in the leaves of Tut beech trees (Figure 6). Respectively, the abundance of the glutathione precursor γGluCys (γEC) that relies on Ser availability for the synthesis of its Cys moiety (Strohm et al., 1995), was also significantly lower in leaves of Tut beech trees.

On the other hand, the lower abundances of sugar-Ps such as (G6P; F6P; M6P) and (G1P; M6P) in Tut compared to Con leaves may indicate lower abundance of sugar-Ps for glycolysis. DHAP was only detectable in spring buds and summer leaves, and tended to be lower in beech trees of the Tut site. Although pyruvate, the product of glycolysis, was lower in Tut compared to Con in summer leaves, the level of citrate synthesized after pyruvate decarboxylation from acetyl-CoA and oxaloacetate was higher. Two TCA-cycle metabolites synthesized downstream of citrate, i.e., malate and fumarate (Supplementary Figure S6), were lower in Tut than in Con leaves. Down-regulation of carbohydrate degradation by glycolysis and the TCA-cycle may be concluded and is supported by reduced abundance of several AAs synthetized from intermediates of glycolysis and the TCA-cycle (Ala, Thr, Arg) for the beech leaves of the Tut forest (Figure 6, Supplementary Figure S7).

On the other hand, sugar-Ps such as (G1P; M6P) and pyruvate are precursors for lipid formation (Supplementary Figure S8). Several non-phospholipids are enhanced in Tut compared to Con leaves that are SQDG, GlcADG, and ASG, which replace phospholipids at low P availability (Okazaki et al., 2013; Lambers et al., 2015; Siebers et al., 2015). The storage lipids TAG, DAG, and their precursor glycerol-3P (Gly3P) showed higher abundance in Tut compared to Con leaves during summer.

##### Bark and wood

During summer, the polar metabolome and lipidome were largely similar for the twig-bark of Tut and Con beeches with exceptions of several N compounds (Figure 6). The AAs Arg (3.6-fold), His (5.4-fold), Asn (14.3-fold), and Trp (20.7-fold) and the Cys precursor O-acetylSer (1.4-fold) were higher in the bark of Con than of Tut beeches.

In the twig-wood the polar metabolome and the lipidome revealed strong differences between the two study sites (Figure 6). However, differences were not observed for sugar-Ps and metabolites of glycolysis, but for TCA-cycle intermediates (Figure 6). Lower malate, cis/trans-aconitate and citrate abundances were found in the twig-wood of Tut beeches that might indicate downregulation of the TCA-cycle. This view is supported by lower levels of nucleotides. Lower levels of 5-aminoimidazole-4-carboxamide ribonucleotide (AICAR) and AMP known to constitute activators of the “cell energy sensor” AMPK (AMP activated protein kinase; Theodorou et al., 1991; Hardie, 2011) support this assumption. The lipid composition of the wood showed strong differences between both forest sites, which were, however, not uniform. Higher abundances of PG and DGDG, but lower levels of PE, lysoPC and of the non-phospholipids GlcADG and GlcCER were detected in the wood of Tut compared to Con beeches.

## Discussion

**Figure 7:**
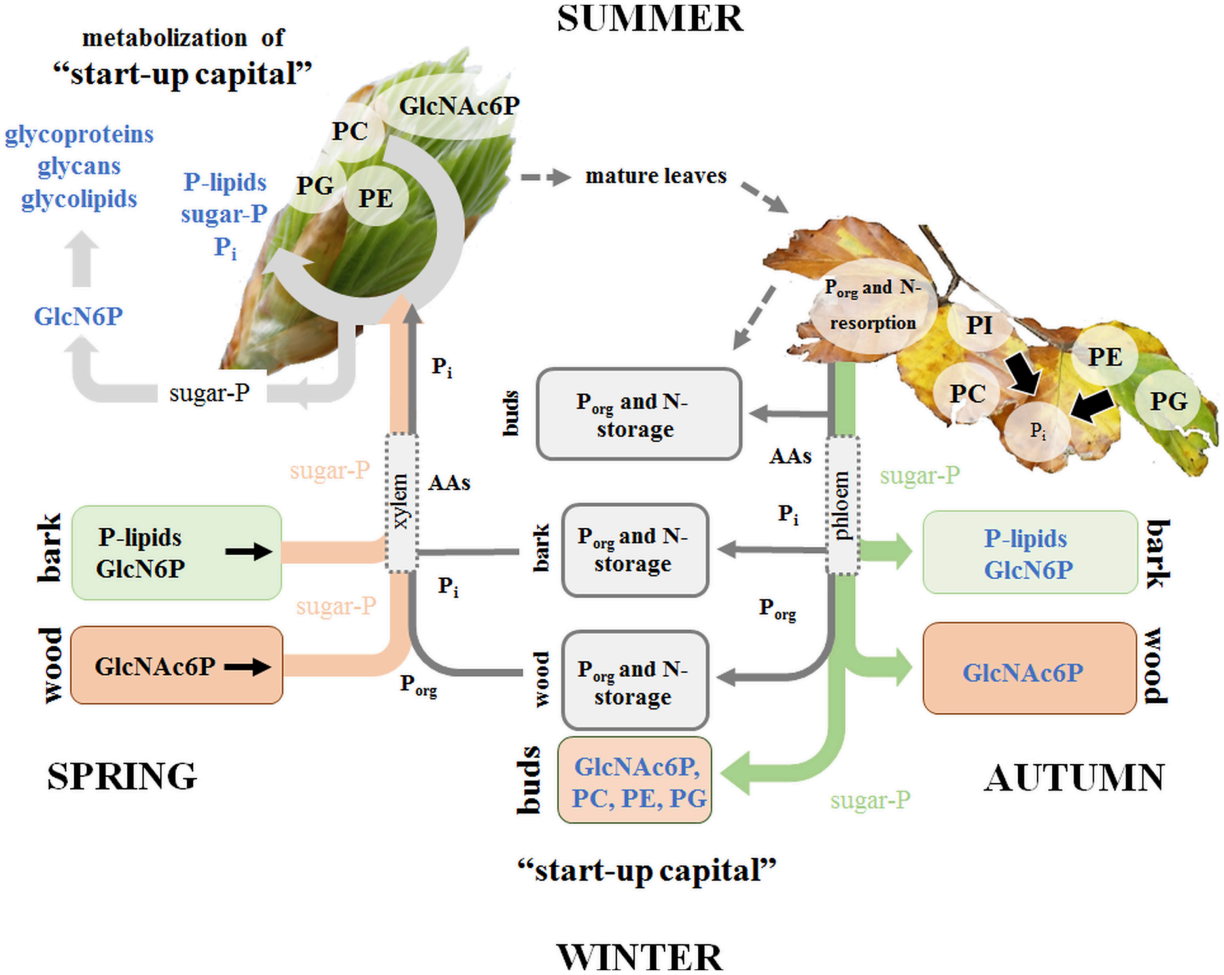
P_org_ degradation in the stem contributes to fulfill P_i_ requirements for leaf development in spring. P and N storage, mobilization and fluxes are presented in gray. Storage and fluxes of distinct compounds of the polar (P) metabolom and (P) lipidome are presented in color. P mobilization in spring for the supply of P to developing buds, which require P_i_ for the energy metabolism and cell proliferation is achieved by three processes. (i) Phospholipid and GlcN6P degradation in the bark as well as GlcNAc6P mobilization in the wood; (ii) subsequent transport of released sugar-Ps and P_i_ to developing buds; and (iii) the use of the phospholipid “start-up capital” in buds built during autumn and/or dormancy. In the buds, GlcN6P can be synthesized from sugar-Ps and serves as a precursor for GlcNAc6P synthesis that can then be used in glycolipids, glycan and glycoprotein synthesis in spring buds (Furo et al., 2015). In autumn, phospholipid degradation and removal of P_i_ from senescent leaves take place prior leaf abscission. P_i_ is subjected to phloem transport and the formation of P-storage pools in bark and wood. In addition, P_i_ is used to build the P “start-up capital” in newly established buds. P_i_ supply of newly established buds in autumn is achieved by both, phloem and xylem transport. The dimension of arrows indicates the estimated contribution of either xylem or phloem transport in spring or autumn.

This study provides a detailed view on the dynamics of P metabolism of the temperate climax forest tree species *F. sylvatica* during annual growth. It could be showed (i) that the profile of polar P metabolites and phospholipids is modulated by the season in leaves, bark and wood (hypothesis 1) for P storage and mobilization, and (ii) that these variations are linked to changes of central metabolic pathways (hypothesis 3). Phospholipids and GlcN6P in the bark, but solely GlcNAc6P in the wood as well as phospholipid accumulation in dormant leaf buds are the hallmarks of P storage during dormancy (Figure 7). The accumulated phospholipids in dormant buds provide the P “start-up capital” for shoot outgrowth in spring. Limitation of soil-P_i_ availability affected phospholipid storage and mobilization, thereby economizing P use in beech twigs *via* several processes (Figure 7). (i) P_i_ availability of leaves was economized in summer by diminished abundance of sugar-Ps and by phospholipid replacement (hypothesis 2). (ii) P resorption from phospholipids of senescent leaves in autumn and accumulation of phospholipids in leaf buds during dormancy was enhanced. (iii) Phospholipid storage in the twig-bark during dormancy was improved. The latter processes complemented the P “start-up capital” for shoot outgrowth in spring at low soil-P_i_ availability. Altogether, the results showed that seasonal changes of polar P metabolites and phospholipids and consequently the internal P-cycling efficiency were affected by the P_i_ availability in the soil (hypothesis 3). Thus, growth restriction by soil-P_i_ limitation was prevented in beech trees by economizing P_i_ availability in leaves and by efficient storage and mobilization of phospholipids.

### Changes in polar metabolite and lipid composition indicate annual P (re)cycling

#### Autumn and winter: P resorption from beech leaves and P storage in twig-bark

The removal of nutrients from senescent leaves and storage pool formation in the stem constitutes a special feature of deciduous trees to restrict loss of nutrients by leaf abscission (Rennenberg and Schmidt, 2010). As a consequence, N (Millard and Grelet, 2010; Wildhagen et al., 2010), S (Herschbach et al., 2012), and P contents (Chapin and Kedrowski, 1983; Keskitalo et al., 2005; Netzer et al., 2017) decrease in leaves and increase in stem tissues during autumn. Mobilization of nutrients from leaves during senescence requires metabolic remodeling to generate phloem mobile metabolites for its transport into storage tissues. Consistently, many proteinogenic AAs increased in senescent compared to summer leaves of adult beech trees (Figure 2). Simultaneously, AAs showed greater abundance in phloem exudates during autumn than in spring (Figure 3). The bark functions as sink organ for phloem-derived AAs and is the site of storage protein formation in autumn (Coleman et al., 1991; Millard and Grelet, 2010; Wildhagen et al., 2010). This is consistent with increased N in twig-bark and wood of beech trees in winter (Figure 1). Particularly prominent, the organic P compounds involved in P mobilization, phloem transport, and storage in the bark were identified in the present study. In beech leaves, phospholipids specifically PG and PI declined in autumn and, thus, constitute P_i_ sources. PG and PI degradation was supported by the increased abundance of lysoPC, an indicator of phospholipid degradation in senescent leaves (Nakamura, 2013; Boudière et al., 2014; Siebers et al., 2015). Consistently, in the arctic tundra forest tree *Betula papyrifera*, the phospholipid pool of leaves decreased by 50% and the nucleic acid P pool by 61% until senescence, thereby explaining 74% of the P resorption from birch leaves during senescence (Chapin and Kedrowski, 1983).

Depending on source-sink relations, resorbed and phloem transported nutrients are allocated to the roots, developing tissues such as apical meristems, to newly established buds or to stem tissues to fill up storage pools (Lough and Lucas, 2006; Lucas et al., 2013). Notably, sugar-Ps were identified as P_org_ compounds transported in the phloem and thereby mediating accumulation of P_org_ in beech buds during dormancy (Netzer et al., 2017). In dormant beech buds, however, phospholipids (PC, PE, PG) and GlcNAc6P rather than sugar-Ps contribute to P_org_ accumulation. Another sink of phloem allocated sugar-Ps are the twig-bark and wood that both accumulated P_org_ during dormancy (Netzer et al., 2017). In the bark, but not in the wood PC, PE, and PG contribute to P_org_ storage (Figures 4, 5). In birch, P storage in stems during dormancy was mediated by nucleic acid-, lipid-, and ester-P (Chapin and Kedrowski, 1983). Hence, further P metabolites such as inositol-6-phosphate known as a common storage compound in grains may furthermore contribute to the P_org_ storage pool as described for poplar (Kurita et al., 2017). Development of an adequate and sophisticated technique for the analysis of IP6 in tree tissues needs further studies (Wu et al., 2009). Neither the extraction method of Lisec et al. (2006) nor a GC-MS approach (Du et al., 2016) nor a common photometric test using the “Ward-reagent” (Vaintraub and Lapteva, 1988; Konieczynski and Wesolowski, 2014) were suitable to quantify IP6 in beech tissues (data not shown). Nevertheless, the present study identified further P_org_ storage compounds; specifically GlcN6P in the bark and its acetylation product GlcNAc6P in the wood (Figures 4, 5). GlcNAc6P, a precursor of glycolipid synthesis (Furo et al., 2015), was highest during dormancy and coincided with highest levels of the galactolipids DGDG and MGDG in beech bark (Figure 4). MGDG and DGDG are uncharged galactolipids and main constituents of the lipid bilayer matrix in thylakoid membranes (Shimojima and Ohta, 2011; Kobayashi, 2016). It may be speculated that increasing DGDG and MGDG play a role in chloroplasts development of bark parenchyma cells for photosynthesis. Bark photosynthesis is a phenomenon observed for several perennial plants including beech (Wittmann et al., 2001) and even in winter (Diehl et al., 1993; Aschan and Pfanz, 2003). Alternatively, increasing amounts of DGDG and MGDG may be involved in frost hardening during dormancy and/or in C storage (Yoshida and Sakai, 1973; Wang and Faust, 1988; Moellering et al., 2010).

#### Spring: P mobilization from twig-bark and xylem transport to developing buds

Bud break in spring requires the release of dormancy to provide sufficient amounts of nutrients for energy production and building blocks for leaf growth and development. The present study shows that the decrease of P_org_ and P_tot_ (Figure 1, Netzer et al., 2017) in twig-bark is due to phospholipid degradation, since PC, PE, and PG declined from dormancy to spring (Figure 4). In addition, degradation of GlcN6P in the bark and GlcNAc6P in the wood could contribute to provide P_i_. Hence, twig phospholipids, but also GlcN6P and GlcNAc6P, are strong candidates for supplying P_i_ to the buds after cleavage by phosphatases (Lan et al., 2015; Siebers et al., 2015) and P_i_ allocation *via* xylem transport. P_i_ was the main transport form of P in the xylem sap of beech in spring, but P_org_ still accounted for ~40% of P_tot_ (Netzer et al., 2017). A number of polar P compounds, i.e., sugar-Ps, nucleotides, and beta-NMN, were identified in the xylem sap and thus contributed to the xylem transported P_org_ pool. The diversity of organic P metabolites was lower in the xylem sap compared to leaves, bark and wood (Table 1). This indicates restricted loading of P metabolites into the xylem sap. Mobilization of nutrients from storage pools of the stem and its transport to developing buds in spring was described for several tree species and may be the consequence of temperature restricted nutrient uptake from the soil (Geßler et al., 1998; Rennenberg et al., 2010; Budzinski et al., 2016). Degradation of bark storage proteins (BSP) during spring to release AAs for its allocation to developing buds *via* the xylem sap is a well-known feature of deciduous trees (Schneider et al., 1996; Nahm et al., 2006; Rennenberg et al., 2010) and was also evident in beech trees of the present study (Figure 3).

#### Spring: metabolic remodeling in buds

During spring large amounts of P_org_ especially beta-NMN, sugar-Ps (Figure 3), P_i_ (Netzer et al., 2017), AAs and organic acids in the xylem sap coincided with peak levels of almost all AAs, sugar-Ps, lipids (present study), and P_i_ (Netzer et al., 2017) in beech buds. This identified bursting leaf buds as sinks for xylem derived nutrients. High levels of polar metabolites of glycolysis and the TCA-cycle in bursting buds indicated boosting of central pathways of C metabolism. Simultaneously, catabolic pathways involved in the production of building blocks for leaf growth and development as previously described for oak buds (Derory et al., 2006) are essential. Adequate P_i_ as a prerequisite for almost all anabolic and catabolic reactions in bursting buds can be provided via xylem transport into buds, and/or from *in situ* remobilization from sugar-Ps and phospholipids by phosphatases (Tian and Liao, 2015). The only P_org_ compound that declined in buds until spring and thus constitutes a potential *in situ* source of P_i_ was GlcNAc6P that on the other hand is involved in the synthesis of glycolipids (Supplementary Figure S8, Furo et al., 2015). In bursting beech buds GlcNAc6P degradation went along with increasing GlcN6P, which is produced from F6P and serves as substrate for GlcNAc6P (Furo et al., 2015). Apparently, GlcNAc6P is used for both, glycolipid formation and as a substrate of P_i_ delivery. Thus, consumption of GlcNAc6P in buds during spring to allow leaf expansion might be enable by its regeneration via F6P and GlcN6P. Remarkable, in bursting beech buds sugar-Ps and phospholipids (PC, PE, PG, PI) increased and thus did not function as P_i_ source (Figure 2). Synthesis of phospholipids as major components of photosynthetic membranes in chloroplasts is highly significant in spring to fulfill the increasing need in energy and carbon compounds. Especially PG constitutes an important compound stabilizing photosynthetic membranes to maintain membrane polarity (Boudière et al., 2014; Siebers et al., 2015; Kobayashi, 2016). The increase of the chloroplast lipids DGDG and MGDG (Kobayashi, 2016) in developing buds provided additional evidence of chloroplast membrane formation at the beginning of the vegetation period that fits well with increased abundance of lipid precursors such as Gly3P, (UDP-Glc; UDP-Gal), DAG, and FF acids. Further support comes from positive correlations of phospholipids with ADP, sugar-6Ps, sugar-1Ps, and DHAP and phospholipid precursors specifically (UDP-Glc; UDP-Gal), DAG, CTP, and UDP. In addition, FF acid correlated significantly to each other (Supplementary Figure S11, Supplementary Table S5). Hence, phospholipid synthesis rather than phospholipid degradation to supply free P_i_ is evident in developing beech buds.

### Annual P (re)cycling is determined by soil-P availability

#### Autumn and winter: nutrient resorption from leaves, P accumulation in buds and wood are enhanced in beech trees on P impoverished soil

It has been hypothesized that the lower plant available soil-P, the more pronounced will be tree internal P cycling (Lang et al., 2016). Consistent with this hypothesis, P_i_ resorption from the phospholipids PC, PE, and PG in senescent leaves was enhanced at the low-P (and N) forest site Tut compared to the Con site. Generally, higher nutrient resorption from senescing beech leaves in Tut was indicated by lower abundances of the non-P thylakoid membrane lipids MGDG, DGDG, and SQDG (Kobayashi, 2016), lower chlorophyll and carotenoid contents and lower levels of proteinogenic AAs (Figure 2, Supplementary Figure S4). Enhanced P resorption was, neither reflected by enhanced P_tot_ and P_org_ concentrations in phloem exudates nor in the bark in autumn and dormancy (Netzer et al., 2017). Consistently, similar polar (P)-metabolome profiles were determined for phloem exudates and twig-bark of both, Tut and Con, beeches. Nevertheless, the phospholipids PI, PE and PC were higher in the wood of Tut compared to Con twigs (Figure 6). Apparently, the twig-wood of Tut beeches constitutes a stronger sink for remobilized P from senescent leaves than the twig-wood of Con beeches, thereby contributing to uncouple P nutrition from P availability in the soil (Lang et al., 2016).

Different to P_tot_ and P_org_, proteinogenic AAs in phloem exudates showed a higher abundance in Tut compared to Con twigs in autumn (Figure 6). This implies enhanced N transport into bark and wood probably for storage protein synthesis in Tut beech trees, as previously shown for poplar trees (Coleman et al., 1991; Millard and Grelet, 2010; Rennenberg et al., 2010; Wildhagen et al., 2010). Inconsistently, N storage was only observed in the bark of Con beech trees (Figure 1). The seasonal dynamic and spatial resolution of protein storage in beech bark and wood has not been studied and N allocation to the roots for storage cannot be excluded. The discrepancy of greater P and N resorption from senescent Tut leaves combined with equal P but higher N abundances in phloem exudates in comparison to Con beech twigs can be explained by different processes. First, higher phloem unloading and improved phloem-to-wood transport of phloem allocated P compounds may take place in Tut beeches. Second, P mobilized from senescent leaves may be consumed and stored in newly established buds (see below). Third, phloem allocated P compounds could have been transported to the main trunk and/or to the roots. Fourth, P resorption during senescence may take place prior or after N resorption. Hence, a high temporal resolution of polar (P) metabolome and lipidome analyses is needed to resolve timing of P vs. N resorption from senescent leaves in future studies.

#### Spring: the polar (P) metabolome and lipidome in beech buds indicates adequate P and N supply for leaf development in spring independent from soil properties

Although the Tut forest is limited by soil-P_i_ and soil-N (Rennenberg and Dannenmann, 2015; Prietzel et al., 2016; Netzer et al., 2017), similar abundances of polar metabolites, nucleotides and AAs were detected in buds during spring (Figure 6). Apparently, bursting buds were well prepared for an adequate energy metabolism and synthesis of membrane compounds, proteins, nucleoids, lignin etc. for its outgrowth independent of soil-P_i_ and soil-N availability. While during dormancy the P lipidome in buds differed between the low and the sufficient soil-P/N site, phospholipid abundance in spring was similar in the developing buds (Figure 6). This seems to be achieved by *de novo* phospholipid synthesis combined with sugar-P and P_i_ import *via* the xylem. The greater P “start-up capital” in the buds of Tut beeches was required, because of lower P_i_ levels in the xylem sap and, thus, lower P supply into the bursting buds in Tut compared to Con beeches (Netzer et al., 2017). Thus, formation of a higher P “start-up capital” in Tut buds constitutes a mechanism to avoid P shortage in case of restricted P supply *via* the xylem sap in spring.

### Phosphorus use in tut beech leaves in summer is economized by metabolome and lipidome remodeling

In early summer, when leaf development of beech trees is completed and metabolic steady state can be expected, the lower soil-P availability at the Tut compared to the Con forest was reflected by distinct differences in the P metabolome of mature leaves. Consistent with lower P_i_ and P_org_ levels (Netzer et al., 2017), diminished abundances of sugar-Ps in beech leaves of Tut trees were observed in the present study. The same was observed in leaves and roots of *Arabidopsis* plants and leaves of *Eucalyptus* trees upon P limitation (Warren, 2011; Pant et al., 2015a,b). Enhanced turnover or reduced synthesis of sugar-Ps in the leaves of Tut beeches could be a strategy to buffer cytosolic P_i_ (Veneklaas et al., 2012), either by releasing P_i_ from sugar-Ps, or by reducing sugar-P synthesis (Warren, 2011; Lambers et al., 2015). In Tut leaves, improved P_i_ availability may additionally be achieved through bypassing P_i_ consuming pyruvate production *via* pyruvate kinase (PK) (Plaxton and Tran, 2011). This assumption is supported by the lower abundance of AMP (Figure 6), an activator of the cytosolic pyruvate kinase (PKc) (Huppe and Turpin, 1994). Pyruvate can be produced from PEP either through pyruvate P_i_-dikinase (PPDK) or *via* cytosolic phosphoenolpyruvate carboxylase (PEPCc) thereby synthesizing oxaloacetate that is converted to malate in the next step via malate dehydrogenase (MDH) in the cytosol (Supplementary Figure S6). After malate import into mitochondria, malate functions as a precursor for pyruvate synthesized *via* malic enzyme (ME) and, hence, for acetyl-CoA production without using P_i_. Furthermore, malate in mitochondria itself can serve as a precursor for citrate synthesis in the TCA-cycle via rebuilding oxaloacetate, the acceptor of acetate from acetyl-CoA. This alternative pathway to fill up the TCA-cycle is thought to operate under P_i_ limitation (Plaxton and Tran, 2011; Plaxton and Shane, 2015). At the first view, such an alternative pathway in Tut beech leaves was not supported from the present data, because both, malate and pyruvate, were lower in Tut compared to Con leaves in summer. As PEPCc is inhibited by malate the lower malate content in Tut leaves may prevent down regulation of PEPCc at P limitation (Plaxton and Shane, 2015). On the other hand, the presented data did not distinguish between malate contents in the cytosol and mitochondria. However, the greater citrate accumulation in Tut than in Con leaves may indicate this alternative pathway but may also be caused by a reduced carbon flux through the TCA-cycle.

Alternatively, the lower abundance of pyruvate in Tut leaves can be explained by its enhanced conversion to acetyl-CoA for FF acid production (Troncoso-Ponce et al., 2016) and thus lipid biosynthesis. Although the abundance of FF acids in Tut beech leaves was significantly lower than in Con leaves, greater abundances of TAG and DAG that are synthesized from FF acids were found (Figure 6, Supplementary Figure S8). Both TAG and DAG can function as carbon storage pools at P deficiency and accordingly, were described as markers for P starvation in *Arabidopsis* (Pant et al., 2015a,b). Accumulation of DAG and TAG at P deficiency may be caused by their reduced export from chloroplasts into the cytosol. Antiporters that simultaneously import P_i_ (Rychter and Rao, 2005; Pant et al., 2015a,b) mediate the export of DAG, TAG, and triose phosphates from chloroplasts into the cytosol. Considering the lower P_i_ level in beech leaves of Tut compared to Con trees in early summer (Netzer et al., 2017), reduced DAG and TAG export from chloroplasts could explain their enrichment in Tut leaves. Furthermore, DAG and TAG are precursors for the synthesis of phospholipid replacing lipids such as MGDG, DGDG, SQDG, and GlcADG (Supplementary Figure S8). Gly3P needed in DAG and TAG synthesis was lower in Tut than in Con leaves (Figure 6) and finally, the higher abundance of the non-phospholipids SQDG and ASG in Tut leaves indicate phospholipid replacement (Lambers et al., 2012, 2015; Siebers et al., 2015). Especially SQDG is known to be involved in phospholipid replacement under P limitation to stabilize PSII in thylakoid membranes and thus to maintain photosynthetic capacity, especially at reduced PG abundance (Boudière et al., 2014). Thus, accumulation of SQDG can be taken as a strong hint for the adaptation of Tut beech leaves to low-soil-P_i_ availability. The replacement of phospholipids by non-phospholipids is consistent with lower P_org_ levels in Tut compared to Con leaves in early summer (Netzer et al., 2017). In summary, the present results strongly support the assumption that adequate P_i_ levels in leaves of Tut beech trees were achieved by (i) bypassing the P_i_-consuming pyruvate synthesis via PKc, (ii) economized sugar-P availability, and (iii) phospholipid replacement by non-phospholipids.

## Conclusion

Avoidance of P loss by autumnal leaf abcission was enabled by economized P cycling in the temperate climax forest tree species *F. sylvatica*. This is achieved by phospholipid degradation in senescent leaves and consecutive P_i_ export *via* the phloem. In the bark, phloem derived P_i_ is used to build P storage pools in form of phospholipids and GlcN6P, whereas in the wood solely GlcNAc6P fulfills the same function. During autumn, newly established buds are prepared for spring outgrowth by building a P “start-up capital” consisting of phospholipids and GlcNAc6P based on P_i_ mobilized from senescent leaves or delivered by xylem transport. In spring, buds are additionally supplied with P *via* the xylem sap in form of sugar-Ps, and in form of P_i_ mobilized from phospholipids stored in the bark during dormancy. P_i_ and sugar-P transported to the buds are channeled into energy metabolism and used for the synthesis of cellular metabolites, such as nucleotides, phospholipids etc., which are needed for growth and development. These processes are essential to economize tree-internal P cycling and are stimulated in trees growing on P impoverished soil. Under these conditions, P use in leaves is economized to establish adequate P_i_ abundance via phospholipid replacement through galacto- and sulfolipids.

## Author contributions

FN did most of the research including tissue sampling in the field campaigns, sample preparation, data analyses, and the preparation of the figures. AO, YO, and KS performed lipid and metabolite analyses. DD performed the IRMS measurements. CH and HR designed the research and provided suggestions during data analyses. CH, FN, and HR wrote the manuscript.

### Conflict of interest statement

The authors declare that the research was conducted in the absence of any commercial or financial relationships that could be construed as a potential conflict of interest. The reviewer AJV and handling Editor declared their shared affiliation, and the handling Editor states that the process nevertheless met the standards of a fair and objective review.

## Supplementary material

Supplementary Table S1 Polar metabolites identified in buds/leaves, bark, wood, xylem sap, and phloem exudates.

Supplementary Table S2 List of all lipid classes identified in buds/leaves, bark, and wood.

Supplementary Table S3 Top 10 polar metabolites and lipids contributing to the differentiation in axis directions in the PLS-DA plots. Data are taken from PLS-DA analyses of beech tissues presented in Supplementary Figures S2, S3. Mean values [*n* = 3 for polar (P) metabolites], (n = 5 for lipids) of the VIP scores of all axis dimensions (X, Y, Z-dimension) are presented.

Supplementary Table S4 Relative contribution of polar P metabolites or phospholipids to the overall separation in the PLS-DA plots. The relative contribution of P metabolites or phospholipids in the VIP score was calculated by summing up VIP score values of all polar P metabolites and phospholipids obtained from the respective PLS-DA analyzes presented in Supplementary Figures S2, S3.

Supplementary Table S5 Pearson's correlation coefficients derived from of correlation analyses.

Supplementary Figure S1 Distribution pattern of polar (P) metabolites in beech twig organs/tissues. Venn diagrams of all identified polar metabolites **(A)** and polar P metabolites **(B)** (irrespective of seasonal differences) in twig organs/tissues, i.e., buds/leaves, bark, wood, xylem sap, and phloem exudate of adult beech trees from the Con (first number) and Tut (second number) forest. A detailed overview of all polar (P) metabolites in the twig tissues is provided in Table 1 and Supplementary Table S1.

Supplementary Figure S2 Differentiation of the lipidome and the polar metabolome of organs/tissues by season and site. PLS-DA score plots of the lipidome **(A,C,E)** and the polar metabolome **(B,D,F)** for buds/leaves **(A,B)**, bark **(C,D)** and wood **(E,F)** of adult Tut (T) and Con (C) beech trees in October (O), February (F), April (A), and June (J). PLS-DA plots of the lipidome and polar metabolome were build using all identified compounds (Supplementary Table S1). The top 10 polar metabolites and lipids contributing to the separation in axis directions of the PLS-DA score plots are presented in the Supplementary Table S3. Statistical analyses were performed with MetaboAnalyst 3.0 (http://www.metaboanalyst.ca/, Xia et al., 2015).

Supplementary Figure S3 Differentiation of the polar metabolome in xylem sap and phloem exudate by season and site. PLS-DA score plots of the polar metabolome of the xylem sap **(A)** and phloem exudates **(B)** collected from twigs of adult beech trees at the Tut (T) and the Con (C) forest in October (O) and April (A). All identified metabolites were included in the PLS-DA analyses and are presented in the Supplementary Table S1. The top three P metabolites causing the separation in the PLS-DA score plots are (i) for the xylem sap: beta-NMN, (T6P; L1P; Suc6P) and GlcNAc6P and (ii) for phloem exudates: (G6P; F6P; M6P), beta-NMN, dTMP. Statistical analyses were done with MetaboAnalyst 3.0 (http://www.metaboanalyst.ca/, Xia et al., 2015).

Supplementary Figure S4 Changes in pigment abundance of leaves during annual growth. Chlorophyll **(A,B)** and carotenoids **(C,D)** of Con (**A,C**; black bars) and Tut beech leaves (**B,D**; gray bars) in autumn (October, Oct), winter (February, Feb), spring (April, Apr), and summer (June, Jun). Data represent mean values ± *S.D*. of five replicates. Different minor letters indicate significant differences between seasons at each forest site (One-Way-ANOVA, or Kruskal–Wallis-ANOVA in case that the requirement of normal distribution and/or equal variances was not fulfilled; *p* < 0.05). Asterisks indicate significant differences of a particular metabolite between both field sites during one season at ^*^*p* < 0.05; ^**^*p* < 0.01 (results of Student's *t*-test or Mann–Whitney tests in case that the requirement of normal distribution or homogeneity of variances of the data was not fulfilled).

Supplementary Figure S5 Plant hormone abundance in beech twig organs/tissues during annual growth. GABA, ABA, GA3, tZ, and tZR in buds/leaves **(A,B)**, bark **(C,D)**, wood **(E,F)**, of Con (**A,C,E**; black bars) and Tut beeches (**B,D,F**; gray bars) in autumn (October, Oct), winter (February, Feb), spring (April, Apr), and summer (June, Jun). Data presented are mean values ± *S.D*. of three replicates. Different minor letters indicate significant differences between seasons at each forest site (One-Way-ANOVA, or Kruskal–Wallis-ANOVA in case that the requirement of normal distribution and/or equal variances was not fulfilled; *p* < 0.05). Asterisks indicate significant differences between both forest sites of a particular metabolite during the respective season. Statistics were done by Student's *t-*test or Mann–Whitney tests in case that the requirement of normal distribution or homogeneity of variances of the data was not fulfilled. ^*^*p* < 0.05; ^**^*p* < 0.01.

Supplementary Figure S6 Central metabolic pathways including steps providing P_i_ at P starvation. The pathways were designed based on Kegg pathways http://www.genome.jp/kegg/pathway.html?sess=2764b8338258d6286de91bbebe6faf46). Abbreviations of metabolites: 3PGA, 3-phosphoglycerate; R5P, ribulose-5P, S7P, sedoheptulose-7P; F6P, fructose-6P; F1,6P_2_, fructose-1,6P_2_; G3P, glyceraldehyde-3P; DHAP, dihydroxyacetone-P; PEP, phosphoenolpyruvate; OAA, oxaloacetate. Abbreviations of enzymes: PFK, phosphofructokinase; PK, pyruvate kinase; PEPC, PEPcarboxylase; MDH, malate dehydrogenase; PDH, pyruvate dehydrogenase.

Supplementary Figure S7 Synthesis of amino acids from metabolites of central metabolic pathways. The pathways were designed based on Kegg pathways (http://www.genome.jp/kegg/pathway.html?sess=2764b8338258d6286de91bbebe6faf46). Abbreviations of metabolites: 3PGA, 3-phosphoglycerate; R5P, ribulose-5P, S7P, sedoheptulose-7P; F6P, fructose-6P; F1,6P_2_, fructose-1,6P_2_; G3P, glyceraldehyde-3P; DHAP, dihydroxyacetone-P; PEP, phosphoenolpyruvate; OAA, oxaloacetate. Abbreviations of enzymes: PFK, phosphofructokinase; PK, pyruvate kinase; PEPC, PEPcarboxylase; MDH, malate dehydrogenase; PDH, pyruvate dehydrogenase.

Supplementary Figure S8 Schematic overview of lipid metabolism independent of cellular compartmentation. Phospholipids are given in red, lipids known to be involved in phospholipid replacement in blue. Abbreviations: ASG, acyl steryl glucoside; DAG, diacylglycerol; DGDG, digalactosyldiacylglycerol; FF acid, free fatty acid; GlcADG, glucuronosyldiacylglycerol; GlcCer, glucosylceramide; GlcNAc6P, N-acetyl-glucosamine 6-phosphate; GlcN6P, glucosamine-6-phosphate; Gly3P, glycerol-3-phosphate; lysoPA, lysophosphatidic acid; lysoPC, lysophosphatidylcholine; MGDG, monogalactosyldiacylglycerol; PA, phosphatidic acid; PC, phosphatidylcholine; PE, phosphatidylethanolamine; PG, phosphatidylglycerol; PI, phosphatidylinositol; SG, steryl ester; SQDG, sulfoquinovosyldiacylglycerol; TAG, triacylglycerol. The Figure was created based on Okazaki et al. (2013, 2015), Siebers et al. (2015), Boudière et al. (2014), Hirabayashi and Ichikawa (2002), Guschina et al. (2014), Li-Beisson et al. (2010), Furo et al. (2015), and Kobayashi (2016).

Supplementary Figure S9 Plant hormone abundance in transport tissues of beech twigs during annual growth. Plant hormones in the xylem sap and in phloem exudates of the Con (black bars) and the Tut (gray bars) beech twig organs/tissues. Data present mean values ± *S.D*. of three replicates. Different minor letters indicate significant differences between seasons at each forest site (One-Way-ANOVA, or Kruskal–Wallis-ANOVA in case that the requirement of normal distribution and/or equal variances was not fulfilled; *p* < 0.05). Asterisks indicate significant differences between both field sites of a particular metabolite during the respective season. Statistics were performed with Student's *t-*test or Mann–Whitney tests in case that the requirement of normal distribution of the data was not fulfilled ^**^*p* < 0.01; ^***^*p* < 0.001.

Supplementary Figure S10 beta-NMN in twig organs/tissues of beech trees. Beta-NMN in leaves **(A)**, bark **(B)**, wood **(C)**, xylem sap **(D)**, and phloem exudates **(E)** of beech trees from the Con (black bars) and Tut (gray bars) forest. Data represent mean values ± *S.D*. of three replicates. Different minor letters show significant differences between the seasons per site (results of One-Way-ANOVA, or Kruskal–Wallis-ANOVA in case that the requirement of normal distribution and/or equal variances was not fulfilled; *p* < 0.05). Asterisks indicate significant differences of a particular metabolite between both field sites during the respective season at ^*^*p* < 0.05 (results of Student's *t-*test or Mann–Whitney tests in case that the requirement of normal distribution of the data was not fulfilled).

Supplementary Figure S11 Visualized correlation analyzes.
