# Supplementary material for: Seasonal Alterations in Organic Phosphorus Metabolism Drive the Phosphorus Economy of Annual Growth in F. sylvatica Trees on P-Impoverished Soil
Source: Front Plant Sci. 2018 Jun 6;9:723. doi: 10.3389/fpls.2018.00723 (PMC5998604; doi:10.3389/fpls.2018.00723)
Supplement: Supplementary Table S3 — Top 10 polar metabolites and lipids contributing to the differentiation in axis directions in the PLS-DA plots. Data are taken from PLS-DA analyses of beech tissues presented in Supplementary Figures S2, S3. Mean values [n = 3 for polar (P) metabolites], (n = 5 for lipids) of the VIP scores of all axis dimensions (X, Y, Z-dimension) are presented. [file Table_3.DOCX]

**Supplementary Table S3. Top 10 polar metabolites and lipids contributing to the differentiation in axis directions in the PLS-DA plots.**

Data are taken from PLS-DA analyses of beech tissues presented in **Supplementary Figures 2, 3**. Mean values (n = 3 for poplar (P) metabolites, (n = 5 for lipids) of the VIP scores of all axis dimensions (X, Y, Z- dimension) are presented.

| **polar metabolites** | | | |  | | |  |  |  | | |  | |  | |  | |  |
| --- | --- | --- | --- | --- | --- | --- | --- | --- | --- | --- | --- | --- | --- | --- | --- | --- | --- | --- |
| **rank** | **leaves** | | | **bark** | | | | **wood** | | | | **xylem sap** | | | | **phloem exudates** | | |
| **1** | Pipecolate | | 2.01 | Phe | | | 2.20 | Phe | | 2.20 | | Anthranilatep-Aminobenzoate | | 1.52 | | Choline | | 1.79 |
| **2** | Succinate | | 1.92 | PhenylacetylGly | | | 2.12 | Ser | | 2.14 | | Raffinose | | 1.49 | | Pyruvate | | 1.71 |
| **3** | HomoPro;Pipecolate | | 1.89 | Trigonelline | | | 2.00 | N-AcetylGlu | | 1.97 | | Asn | | 1.48 | | Shikimate | | 1.69 |
| **4** | Arg | | 1.86 | Mugineate | | | 1.97 | Quinate | | 1.95 | | N-Acetylneuraminate | | 1.48 | | Mucic acid | | 1.68 |
| **5** | Arginosuccinate | | 1.83 | Carnitine | | | 1.93 | GABA | | 1.93 | | Met | | 1.46 | | PhenylacetylGly | | 1.68 |
| **6** | Trigonelline | | 1.82 | Quinate | | | 1.92 | 2-Isopropylmalate | | 1.88 | | Phe | | 1.45 | | Pantothenate | | 1.63 |
| **7** | Glycerate | | 1.82 | His | | | 1.92 | Mugineate | | 1.86 | | Val | | 1.45 | | O-AcetylSer | | 1.63 |
| **8** | Thiamine | | 1.82 | GSSG | | | 1.90 | Trigonelline | | 1.84 | | His | | 1.44 | | GSH | | 1.62 |
| **9** | Betonicine | | 1.82 | Succinate | | | 1.87 | Adenosine | | 1.81 | | IsoAsn3-Ureidopropionate | | 1.43 | | Sarcosine | | 1.60 |
| **10** | DOPA | | 1.81 | 4-Pyridoxate | | | 1.86 | Gly | | 1.81 | | Thr | | 1.43 | | GA3 | | 1.57 |
| **lipids** | |  | | |  |  | | | | |  | |  | |  | |  |  |
| **rank** | | **leaves** | | | | **bark** | | | | | | | **wood** | | | |  |  |
| **1** | | DGDG | | | 1.58 | GlcADG | | | | | 2.25 | | SQDG | | 2.08 | |  |  |
| **2** | | Prenylquinone plastoquinone-9 | | | 1.38 | Prenylquinone plastochromanol-8 | | | | | 1.91 | | PE | | 1.94 | |  |  |
| **3** | | LysoPC | | | 1.27 | chlorophyll | | | | | 1.67 | | Prenylquinone alpha-tocopherol | | 1.85 | |  |  |
| **4** | | chlorophyll | | | 1.22 | Polyisoprenoid undecaprenol | | | | | 1.26 | | Prenylquinone plastochromanol-8 | | 1.40 | |  |  |
| **5** | | steryl ester | | | 1.21 | Prenylquinone alpha-tocopherol | | | | | 1.12 | | PI | | 1.16 | |  |  |
| **6** | | PG | | | 1.20 | DGDG | | | | | 1.10 | | Glucosylceramide | | 1.14 | |  |  |
| **7** | | FS beta-sitosterol | | | 1.18 | DAG | | | | | 1.05 | | sinapoyl-choline | | 1.09 | |  |  |
| **8** | | FF acid | | | 1.17 | PE | | | | | 1.03 | | SG | | 0.96 | |  |  |
| **9** | | MGDG | | | 1.17 | GlcCER | | | | | 1.01 | | TAG | | 0.91 | |  |  |
|  | | PC | | | 1.04 | steryl ester | | | | | 0.97 | | chlorophyll | | 0.90 | |  |  |

**Supplementary Table S4. Relative contribution of polar P metabolites or phospholipids to the overall separation in the PLS-DA plots.**

The relative contribution of P metabolites or phospholipids in the VIP score was calculated by summing up VIP score values of all polar P metabolites and phospholipids obtained from the respective PLS-DA analyzes presented in **Supplementary Figures S2 and S3**.

| Relative contribution of the sum of all P-compounds [%] | | | | |
| --- | --- | --- | --- | --- |
| tissue / sap | P- metabolome | | P- lipodome | |
|  | Con | Tut | Con | Tut |
| buds/leaves | 13 | 13 | 19 | 20 |
| bark | 13 | 10 | 12 | 16 |
| wood | 12 | 12 | 18 | 18 |
| xylem sap | 8 | 7 | nd | nd |
| phloem exudates | 7 | 4 | nd | nd |
