# Supplementary material for: Seasonal Alterations in Organic Phosphorus Metabolism Drive the Phosphorus Economy of Annual Growth in F. sylvatica Trees on P-Impoverished Soil
Source: Front Plant Sci. 2018 Jun 6;9:723. doi: 10.3389/fpls.2018.00723 (PMC5998604; doi:10.3389/fpls.2018.00723)

Seasonal alterations in organic phosphorus metabolism drive the phosphorus economy of annual growth in *F. sylvatica* trees on P-impoverished soil

Florian Netzer[¶,1], Cornelia Herschbach[¶,1,3*], Akira Oikawa[2], Yozo Okazaki[2], David Dubbert[3], Kazuki Saito[2,4] and Heinz Rennenberg[1,5]

Supplemental Figures

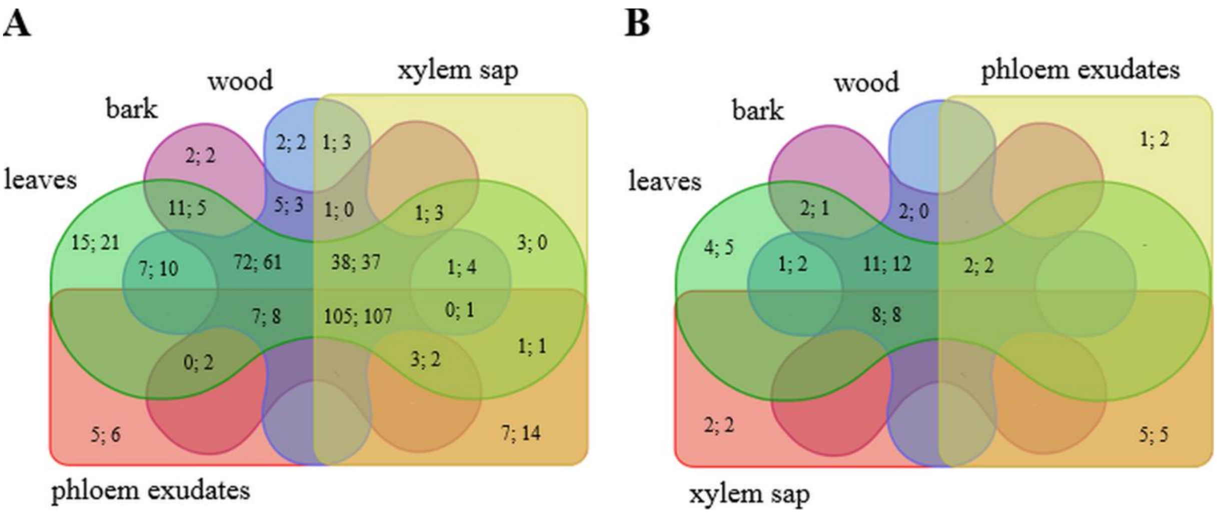

Supplementary Figure S1. Distribution pattern of polar (P) metabolites in beech twig organs/tissues.

Venn diagrams of all identified polar metabolites (**A**) and polar P metabolites (**B**) (irrespective of seasonal differences) in twig organs/tissues, i.e. buds/leaves, bark, wood, xylem sap and phloem exudate of adult beech trees from the Con (first number) and Tut (second number) forest. A detailed overview of all polar (P) metabolites in the twig tissues is provided in **Table 1** and **Supplementary Table S1**.

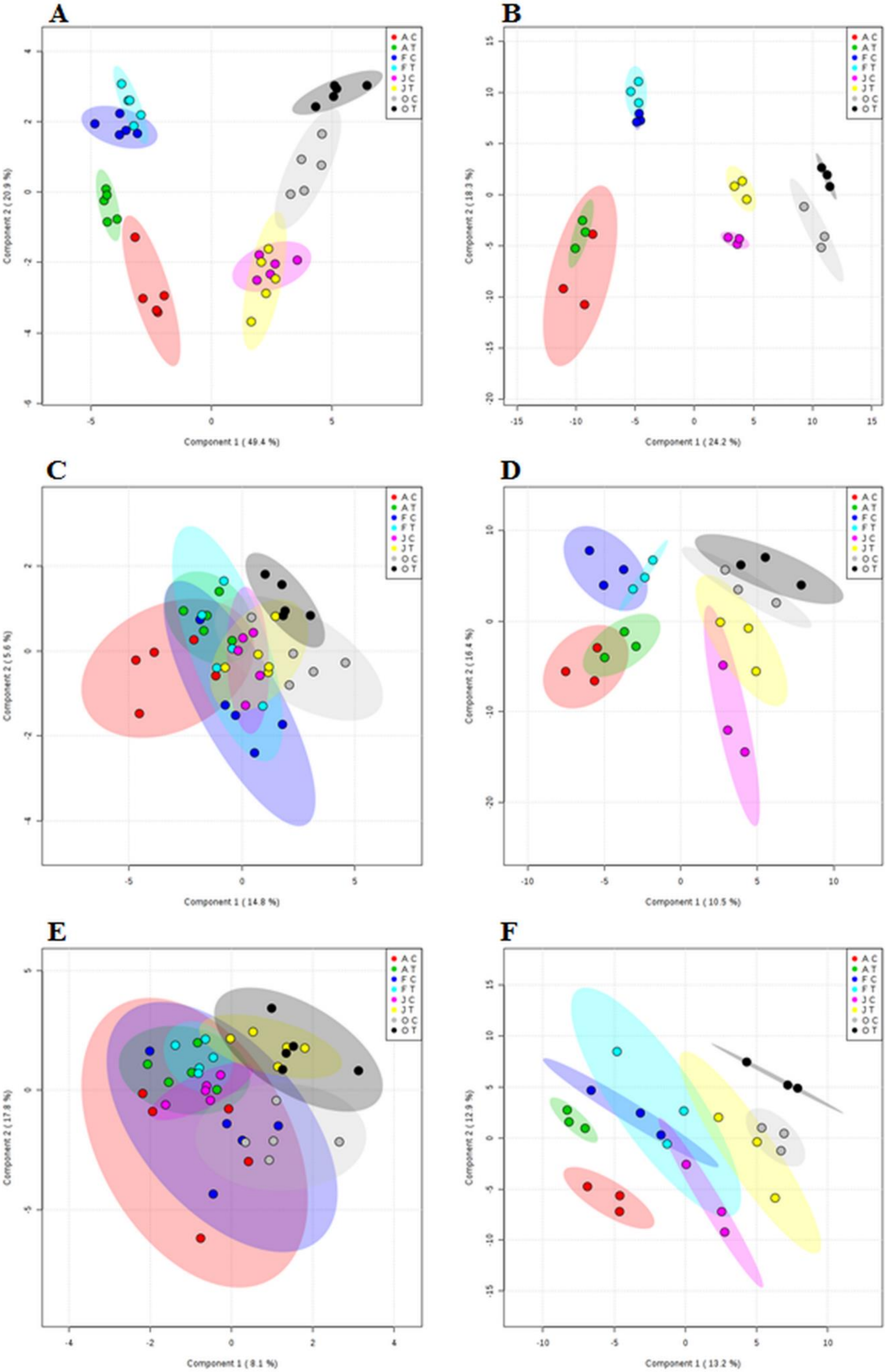
A
Component 1 (49.4 %)
Component 2 (20.9 %)
B
Component 1 (24.2 %)
Component 2 (18.3 %)
C
Component 1 (14.8 %)
Component 2 (5.6 %)
D
Component 1 (10.5 %)
Component 2 (16.4 %)
E
Component 1 (8.1 %)
Component 2 (17.8 %)
F
Component 1 (13.2 %)
Component 2 (12.9 %)
AC
AT
FC
FT
JC
JT
OC
OT

Supplementary Figure S2. Differentiation of the lipidome and the polar metabolome of organs/tissues by season and site.

PLS-DA score plots of the lipidome (**A**, **C**, **E**) and the polar metabolome (**B**, **D**, **F**) for buds/leaves (**A**, **B**), bark (**C**, **D**) and wood (**E**, **F**) of adult Tut (T) and Con (C) beech trees in October (O), February (F) April (A) and June (J). PLS-DA plots of the lipidome and polar metabolome were build using all identified compounds (**Supplementary Table S1**). The top ten polar metabolites and lipids contributing to the separation in axis directions of the PLS-DA score plots are presented in the **Supplementary Table S3**. Statistical analyses were performed with MetaboAnalyst 3.0 (http://www.metaboanalyst.ca/, Xia et al., 2015).

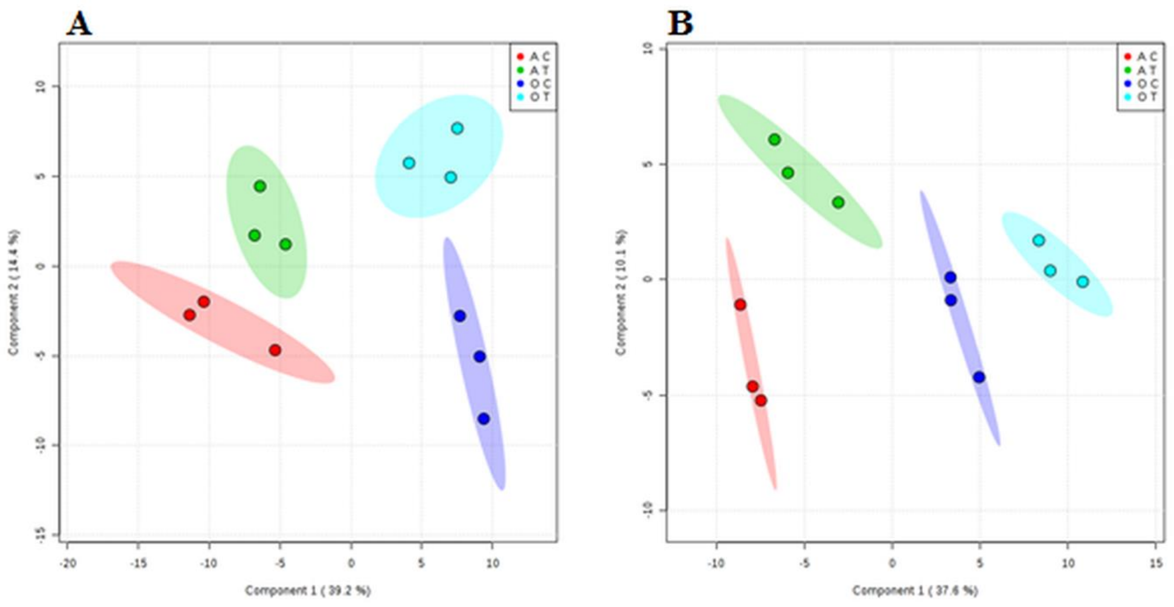

Supplementary Figure S3. Differentiation of the polar metabolome in xylem sap and phloem exudate by season and site.

PLS-DA score plots of the polar metabolome of the xylem sap (**A**) and phloem exudates (**B**) collected from twigs of adult beech trees at the Tut (T) and the Con (C) forest in October (O) and April (A). All identified metabolites were included in the PLS-DA analyses and are presented in the **Supplementary Table S1**. The top three P-metabolites causing the separation in the PLS-DA score plots are (i) for the xylem sap: beta-NMN, (T6P; L1P; Suc6P) and GlcNAc6P and (ii) for phloem exudates: (G6P; F6P; M6P), beta-NMN, dTMP. Statistical analyses were done with MetaboAnalyst 3.0 (http://www.metaboanalyst.ca/, Xia et al., 2015).

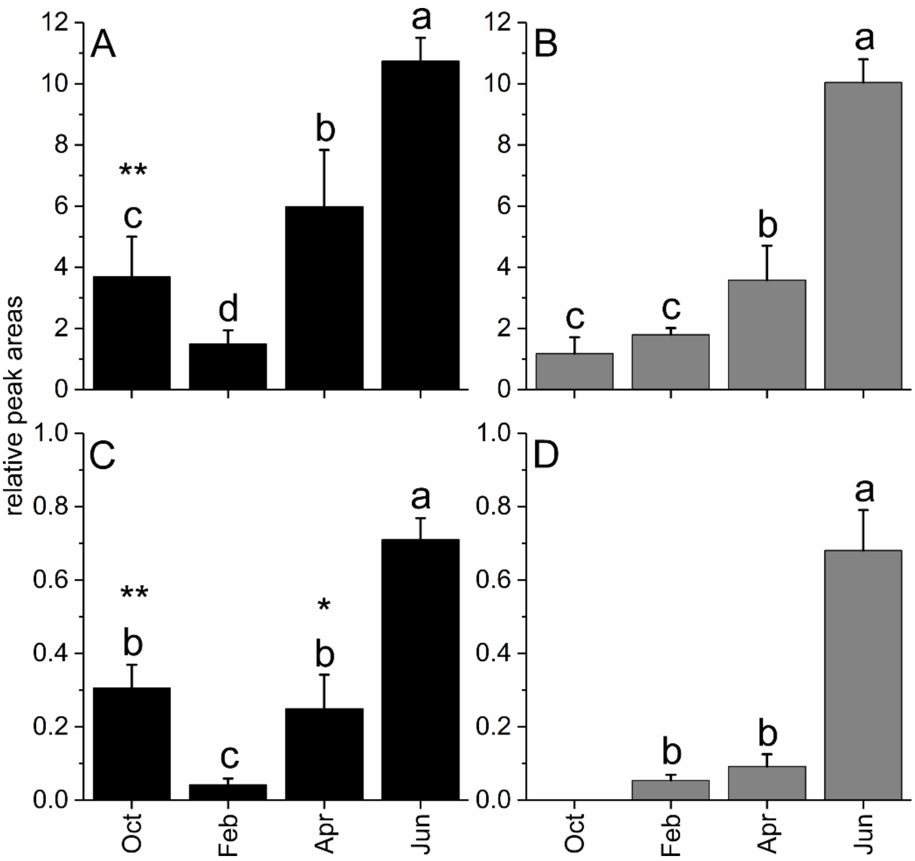

Supplementary Figure S4. Changes in pigment abundance of leaves during annual growth.

Chlorophyll (**A**, **B**) and carotenoids (**C**, **D**) of Con (**A**, **C**; black bars) and Tut beech leaves (**B**, **D**; gray bars) in autumn (October, Oct), winter (February, Feb), spring (April, Apr) and summer (June, Jun). Data represent mean values ± S.D. of five replicates. Different minor letters indicate significant differences between seasons at each forest site (One-Way-ANOVA, or Kruskal-Wallis-ANOVA in case that the requirement of normal distribution and/or equal variances was not fulfilled; $p < 0.05$). Asterisks indicate significant differences of a particular metabolite between both field sites during one season at * $p < 0.05$; ** $p < 0.01$ (results of Student's t-test or Mann-Whitney tests in case that the requirement of normal distribution or homogeneity of variances of the data was not fulfilled).

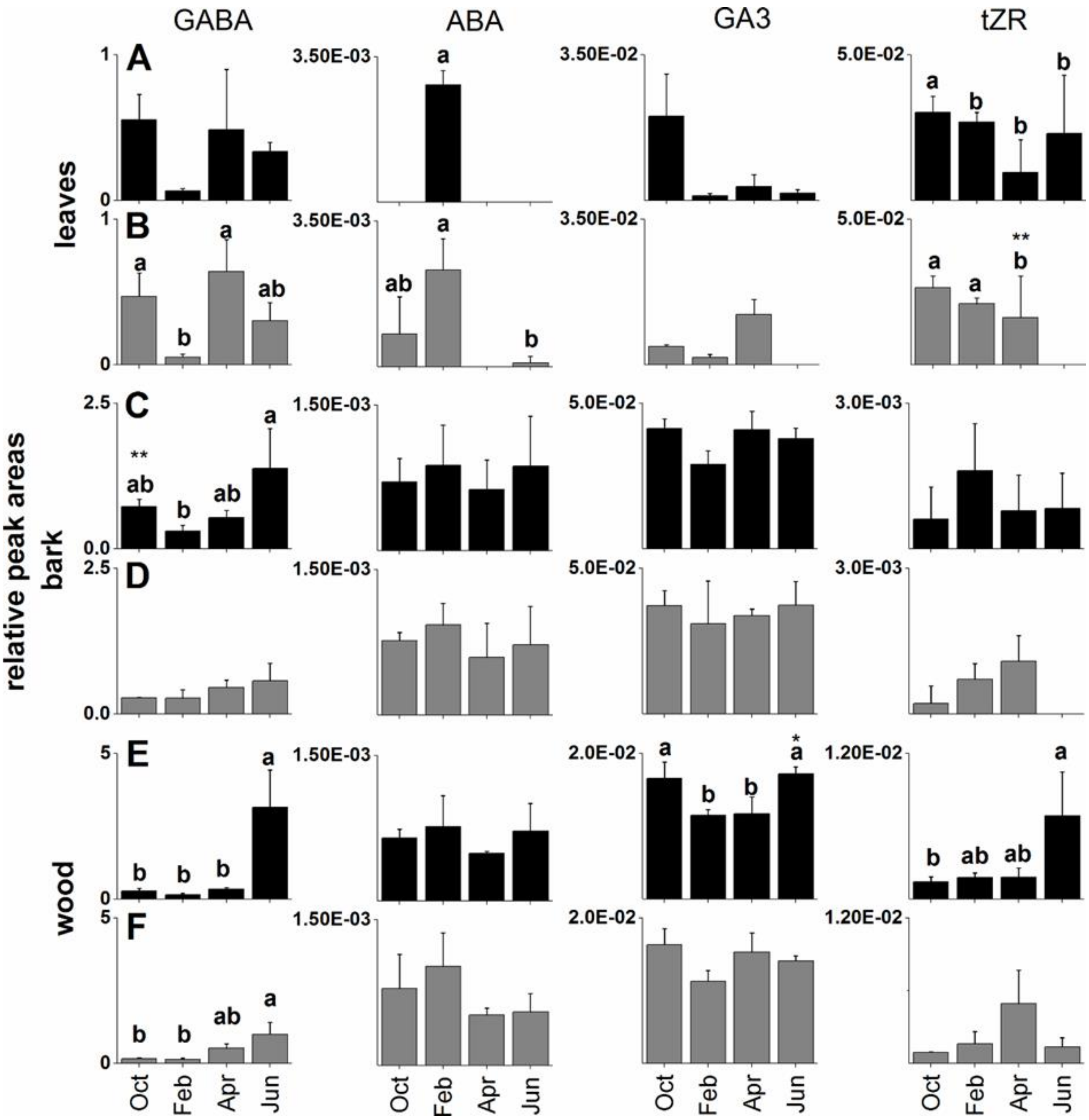

Supplementary Figure S5. Plant hormone abundance in beech twig organs/tissues during annual growth.

GABA, ABA, GA3, tZ and tZR in buds/leaves (**A**, **B**), bark (**C**, **D**), wood (**E**, **F**), of Con (**A**, **C**, **E**; black bars) and Tut beeches (**B**, **D**, **F**; gray bars) in autumn (October, Oct), winter (February, Feb), spring (April, Apr) and summer (June, Jun). Data presented are mean values ± S.D. of three replicates. Different minor letters indicate significant differences between seasons at each forest site (One-Way-ANOVA, or Kruskal-Wallis-ANOVA in case that the requirement of normal distribution and/or equal variances was not fulfilled; $p < 0.05$). Asterisks indicate significant differences between both forest sites of a particular metabolite during the respective season. Statistics were done by Student's t-test or Mann-Whitney tests in case that the requirement of normal distribution or homogeneity of variances of the data was not fulfilled. * $p < 0.05$; ** $p < 0.01$.

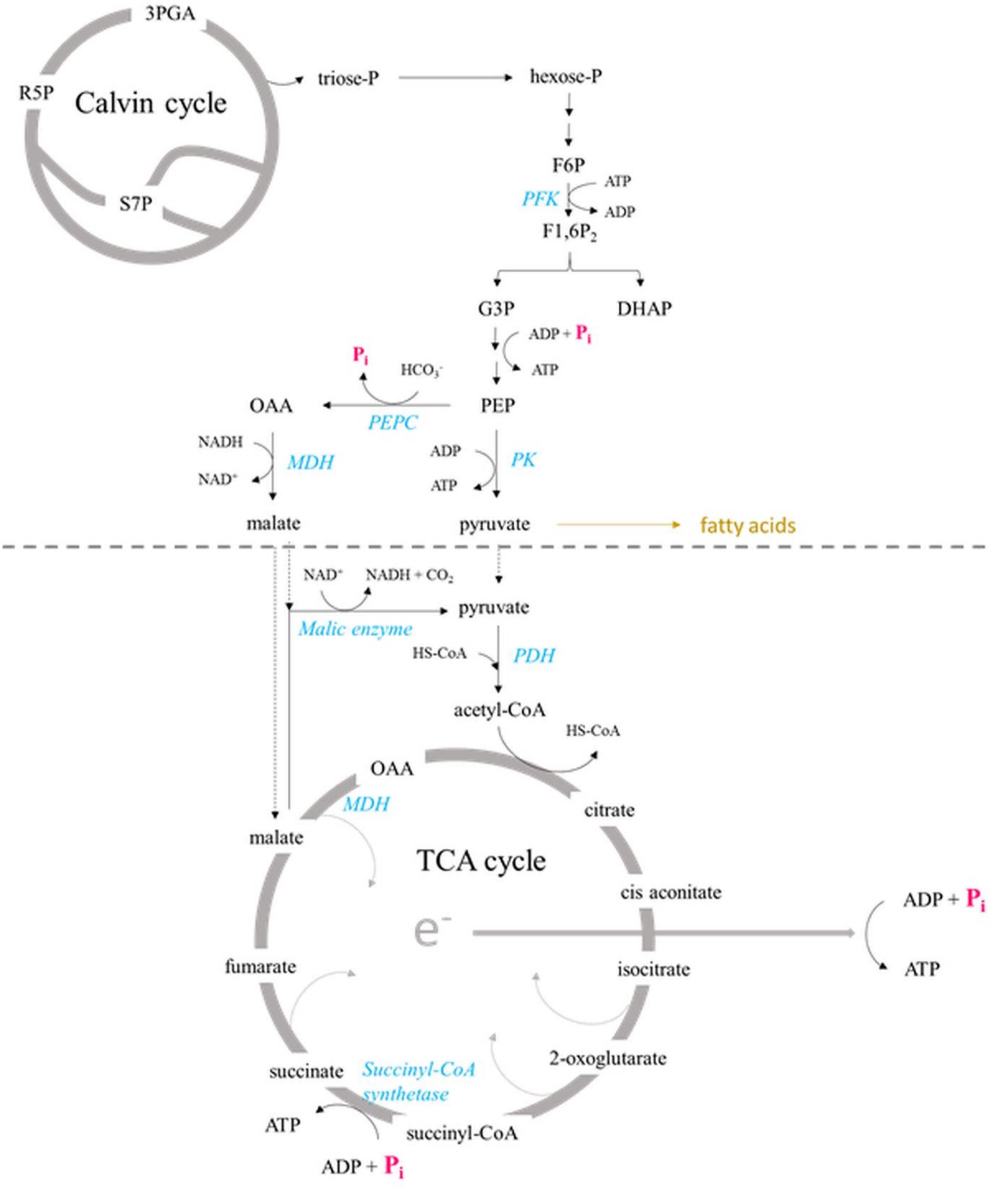

Supplementary Figure S6. Central metabolic pathways including steps providing P_i at P starvation.

The pathways were designed based on Kegg pathways (http://www.genome.jp/kegg/pathway.html?sess=2764b8338258d6286de91bbebe6faf46). Abbreviations of metabolites: 3PGA, 3-phosphoglycerate; R5P, ribulose-5P, S7P, sedoheptulose-7P; F6P, fructose-6P; F1,6P_2, fructose-1,6P_2; G3P, glyceraldehyde-3P; DHAP, dihydroxyacetone-P; PEP: phosphoenolpyruvate; OAA: oxaloacetate. Abbreviations of enzymes: PFK: phosphofructokinase; PK: pyruvate kinase; PEPC: PEPcarboxylase; MDH: malate dehydrogenase; PDH: pyruvate dehydrogenase.

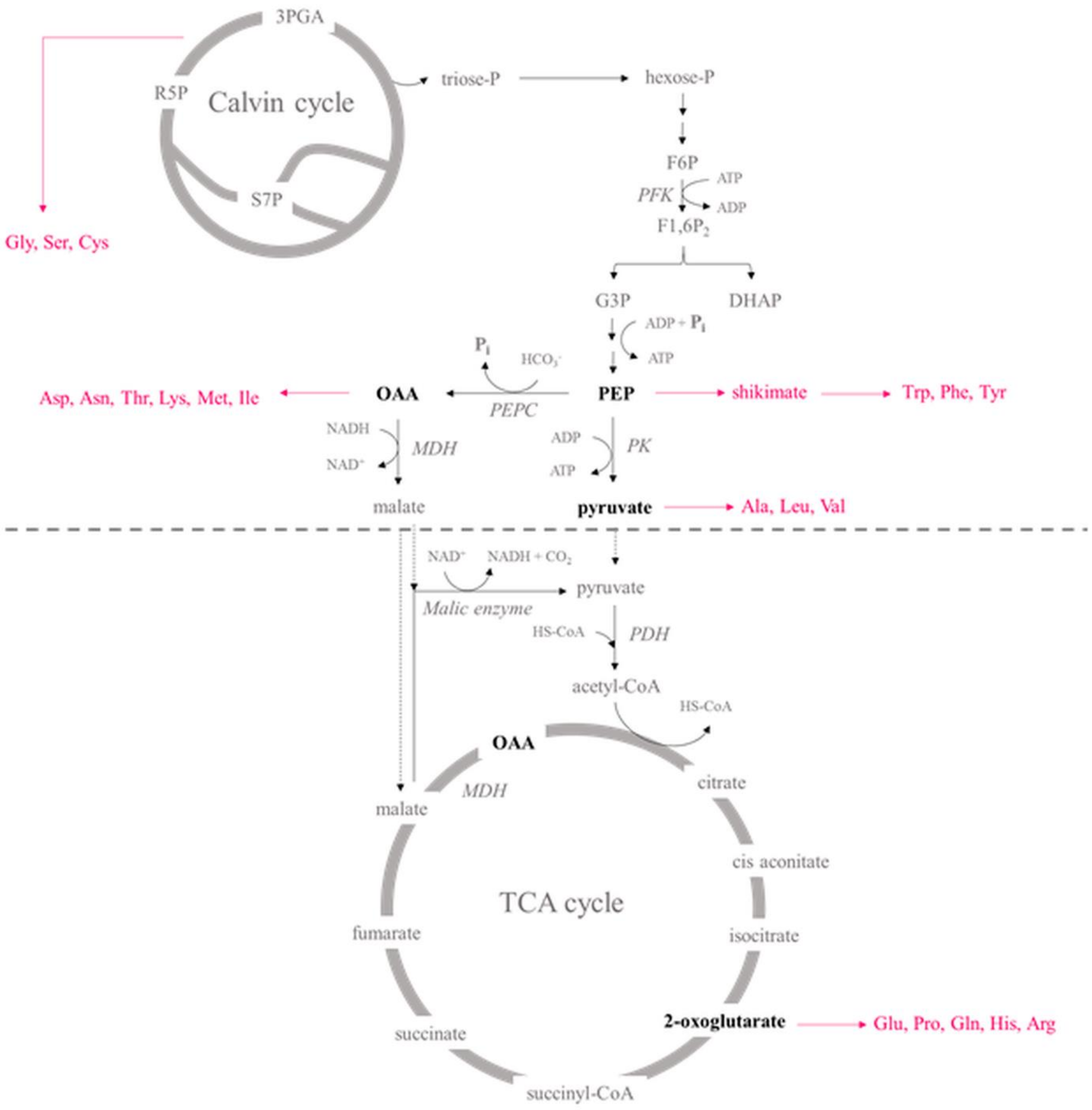

Supplementary Figure S7. Synthesis of amino acids from metabolites of central metabolic pathways.

The pathways were designed based on Kegg pathways (http://www.genome.jp/kegg/pathway.html?sess=2764b8338258d6286de91bbebe6faf46). Abbreviations of metabolites: 3PGA, 3-phosphoglycerate; R5P, ribulose-5P, S7P, sedoheptulose-7P; F6P, fructose-6P; F1,6P_2, fructose-1,6P_2; G3P, glyceraldehyde-3P; DHAP, dihydroxyacetone-P; PEP: phosphoenolpyruvate; OAA: oxaloacetate. Abbreviations of enzymes: PFK: phosphofructokinase; PK: pyruvate kinase; PEPC: PEPcarboxylase; MDH: malate dehydrogenase; PDH: pyruvate dehydrogenase.

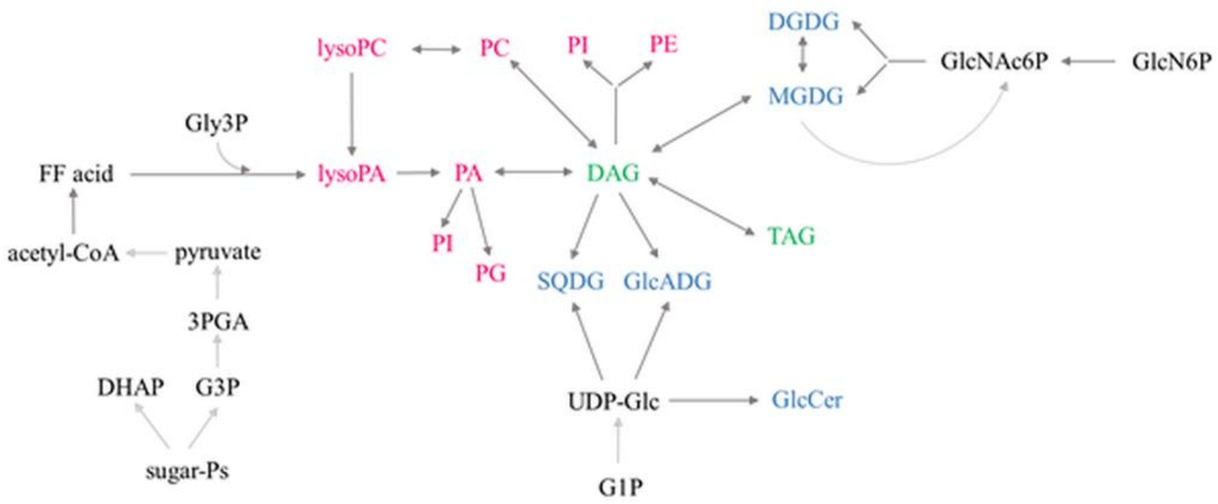

Supplementary Figure S8. Schematic overview of lipid metabolism independent of cellular compartmentation.

Phospholipids are given in red, lipids known to be involved in phospholipid replacement in blue. Abbreviations: ASG, acyl steryl glucoside; DAG, diacylglycerol; DGDG, digalactosyldiacylglycerol; FF acid, free fatty acid; GlcADG, glucuronosyldiacylglycerol; GlcCer, glucosylceramide; GlcNAc6P, N-acetyl-glucosamine 6-phosphate; GlcN6P, glucosamine-6-phosphate; Gly3P, glycerol-3-phosphate; lysoPA, lysophosphatidic acid; lysoPC, lysophosphatidylcholine; MGDG, monogalactosyldiacylglycerol; PA, phosphatidic acid; PC, phosphatidylcholine; PE, phosphatidylethanolamine; PG, phosphatidylglycerol; PI, phosphatidylinositol; SG, steryl ester; SQDG, sulfoquinovosyldiacylglycerol; TAG, triacylglycerol. The Figure was created based on Okazaki et al. (2013, 2015), Siebers et al. (2015), Boudière et al. (2014), Hirabayasi and Ichikawa (2002), Guschina et al. (2014), Li-Beissen et al. (2010), Furo et al. (2015) and Kobayashi (2016).

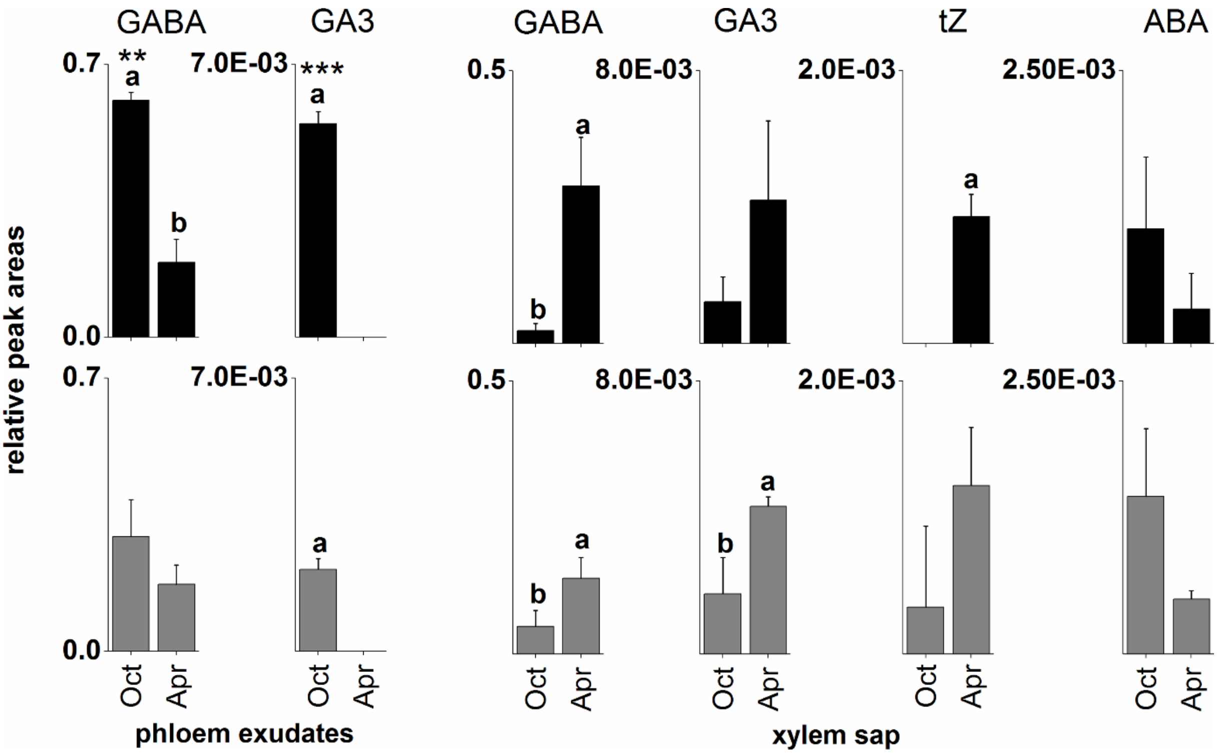

Supplementary Figure S9. Plant hormone abundance in transport tissues of beech twigs during annual growth.

Plant hormones in the xylem sap and in phloem exudates of the Con (black bars) and the Tut (gray bars) beech twig organs/tissues. Data present mean values ± S.D. of three replicates. Different minor letters indicate significant differences between seasons at each forest site (One-Way-ANOVA, or Kruskal-Wallis-ANOVA in case that the requirement of normal distribution and/or equal variances was not fulfilled; $p < 0.05$). Asterisks indicate significant differences between both field sites of a particular metabolite during the respective season. Statistics were performed with Student's t-test or Mann-Whitney tests in case that the requirement of normal distribution of the data was not fulfilled ** $p < 0.01$; *** $p < 0.001$.

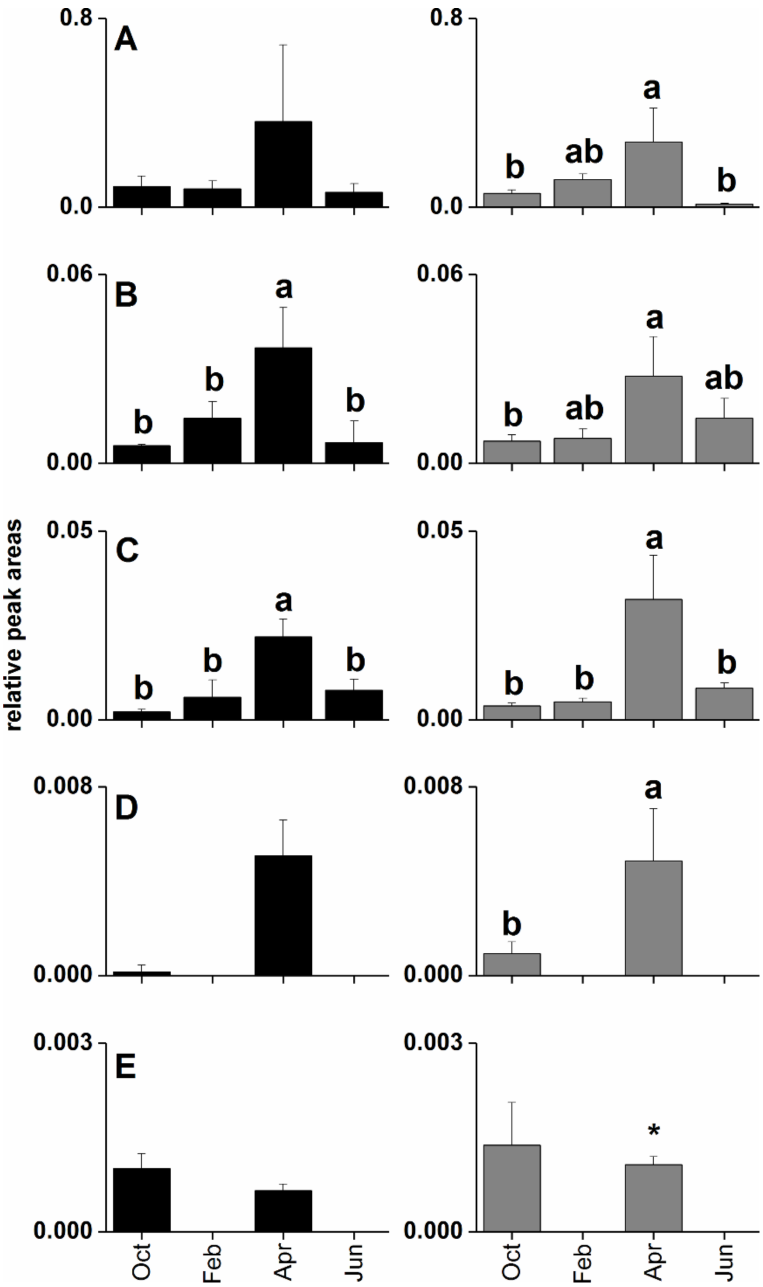

Supplementary Figure S10. beta-NMN in twig organs/tissues of beech trees.

beta-NMN in leaves (**A**), bark (**B**), wood (**C**), xylem sap (**D**) and phloem exudates (**E**) of beech trees from the Con (black bars) and Tut (gray bars) forest. Data represent mean values ± S.D. of three replicates. Different minor letters show significant differences between the seasons per site (results of One-Way-ANOVA, or Kruskal-Wallis-ANOVA in case that the requirement of normal distribution and/or equal variances was not fulfilled; $p < 0.05$). Asterisks indicate significant differences of a particular metabolite between both field sites during the respective season at * $p < 0.05$ (results of Student's t-test or Mann-Whitney tests in case that the requirement of normal distribution of the data was not fulfilled).

S11 Fig. Visualized correlation analyzes.

A: amino acid metabolism in leaves

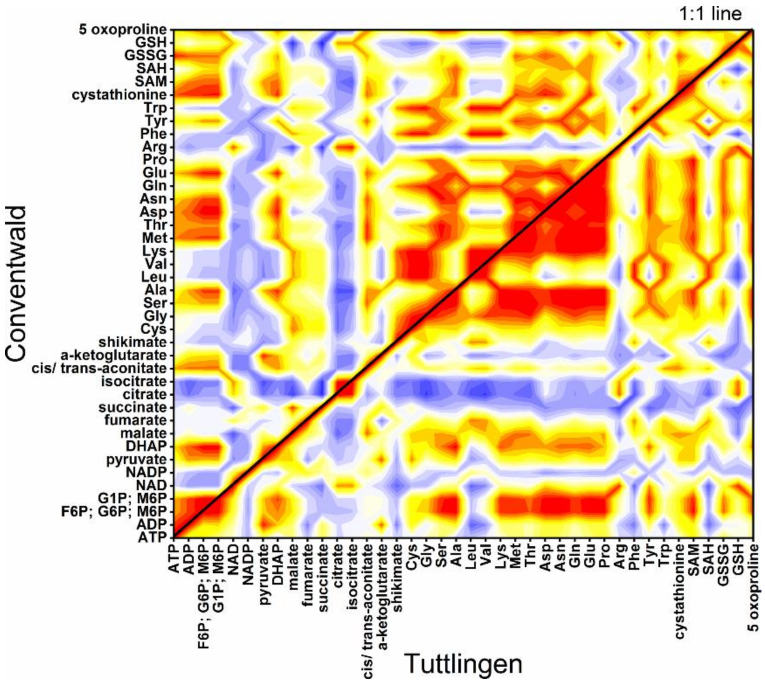

B: amino acid metabolism in wood

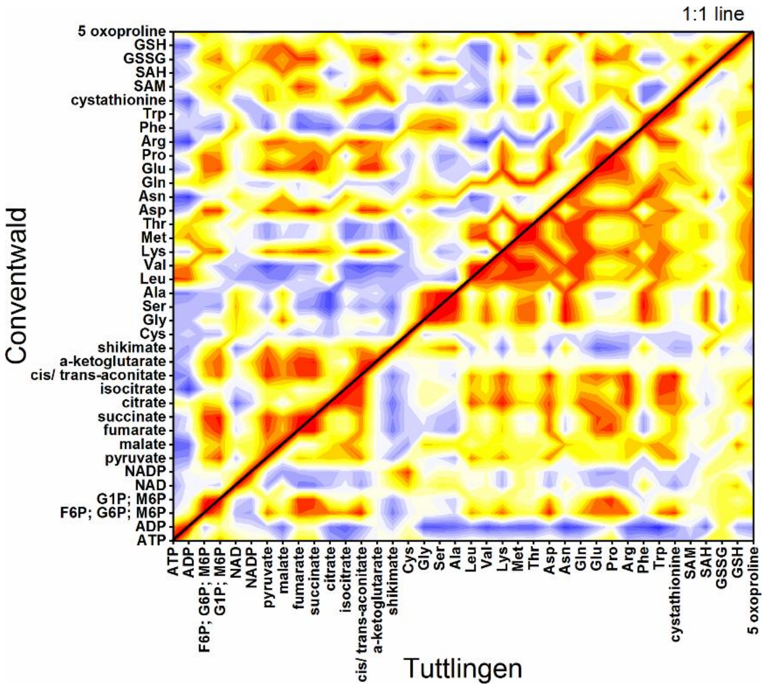

C: purine metabolism in leaves

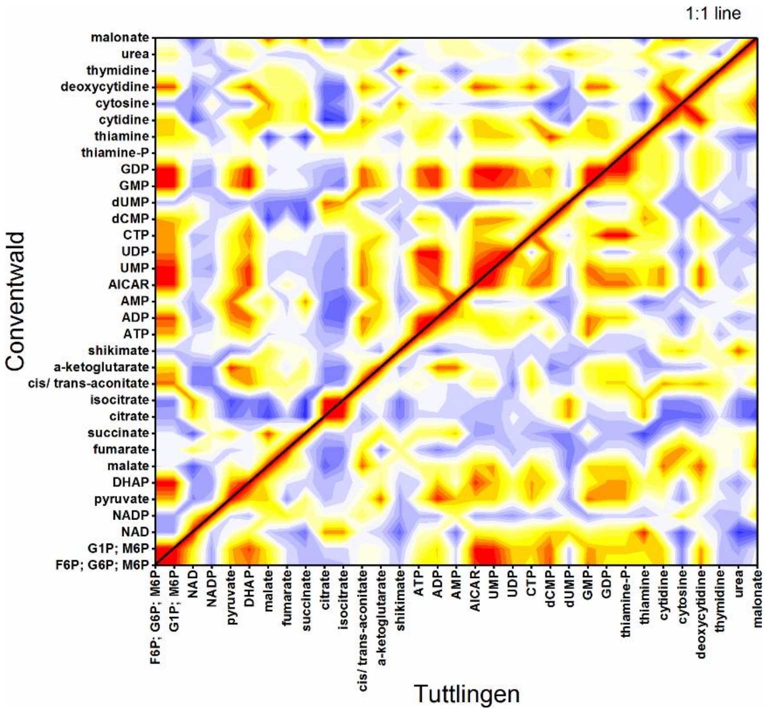

D: purine metabolism in wood

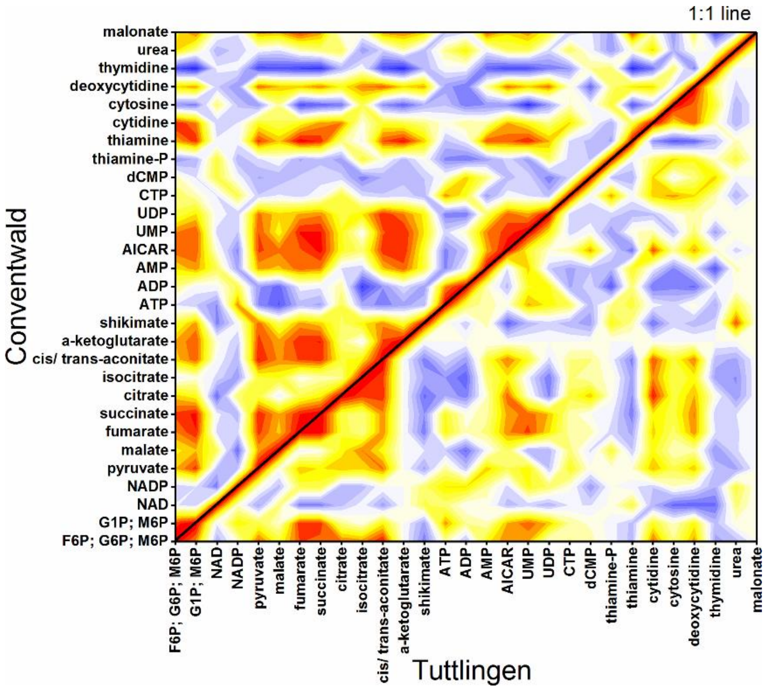

E: lipid metabolism in leaves

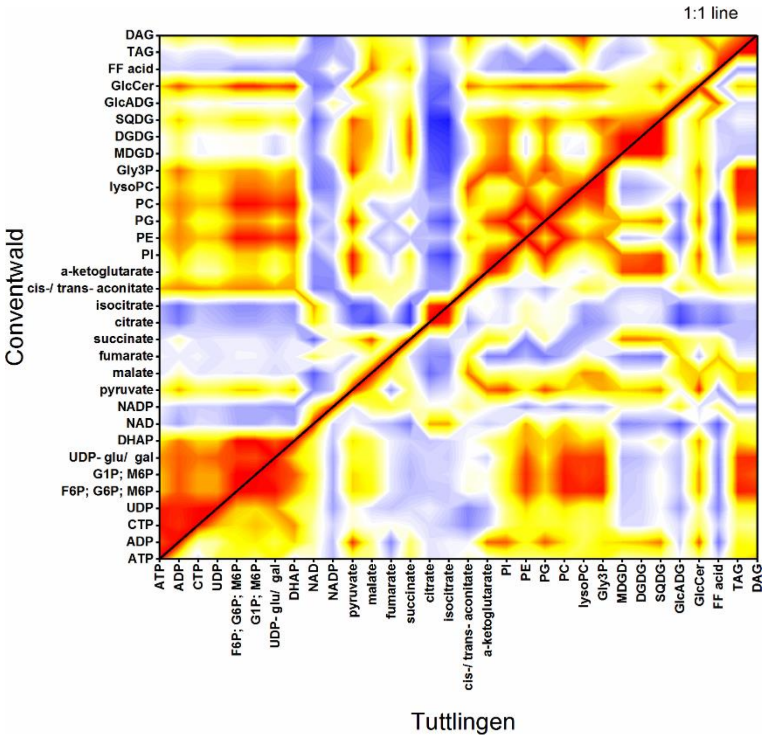

F: lipid metabolism in wood

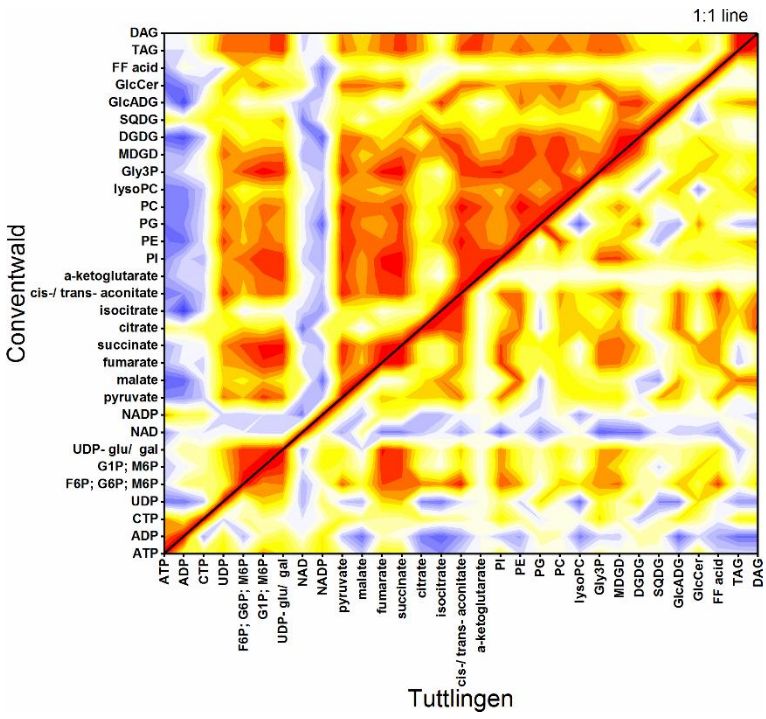

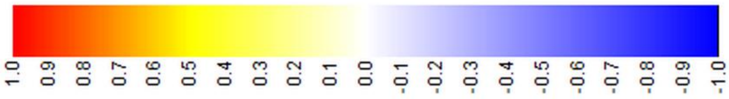

Supplement: Supplementary Figure S1 — Distribution pattern of polar (P) metabolites in beech twig organs/tissues. Venn diagrams of all identified polar metabolites (A) and polar P metabolites (B) (irrespective of seasonal differences) in twig organs/tissues, i.e., buds/leaves, bark, wood, xylem sap, and phloem exudate of adult beech trees from the Con (first number) and Tut (second number) forest. A detailed overview of all polar (P) metabolites in the twig tissues is provided in Table 1 and Supplementary Table S1. [file Data_Sheet_1.pdf]
